# Removing the Bottleneck: Introducing cMatch - A Lightweight Tool for Construct-Matching in Synthetic Biology

**DOI:** 10.3389/fbioe.2021.785131

**Published:** 2022-01-10

**Authors:** Alexis Casas, Matthieu Bultelle, Charles Motraghi, Richard Kitney

**Affiliations:** Department of Bioengineering, Imperial College London, London, United Kingdom

**Keywords:** synthetic biology, tool, software, parts, genetic construct, matching, quality control

## Abstract

We present a software tool, called cMatch, to reconstruct and identify synthetic genetic constructs from their sequences, or a set of sub-sequences—based on two practical pieces of information: their modular structure, and libraries of components. Although developed for combinatorial pathway engineering problems and addressing their quality control (QC) bottleneck, cMatch is not restricted to these applications. QC takes place post assembly, transformation and growth. It has a simple goal, to verify that the genetic material contained in a cell matches what was intended to be built - and when it is not the case, to locate the discrepancies and estimate their severity. In terms of reproducibility/reliability, the QC step is crucial. Failure at this step requires repetition of the construction and/or sequencing steps. When performed manually or semi-manually QC is an extremely time-consuming, error prone process, which scales very poorly with the number of constructs and their complexity. To make QC frictionless and more reliable, cMatch performs an operation we have called “construct-matching” and automates it. Construct-matching is more thorough than simple sequence-matching, as it matches at the functional level-and quantifies the matching at the individual component level and across the whole construct. Two algorithms (called CM_1 and CM_2) are presented. They differ according to the nature of their inputs. CM_1 is the core algorithm for construct-matching and is to be used when input sequences are long enough to cover constructs in their entirety (e.g., obtained with methods such as next generation sequencing). CM_2 is an extension designed to deal with shorter data (e.g., obtained with Sanger sequencing), and that need recombining. Both algorithms are shown to yield accurate construct-matching in a few minutes (even on hardware with limited processing power), together with a set of metrics that can be used to improve the robustness of the decision-making process. To ensure reliability and reproducibility, cMatch builds on the highly validated pairwise-matching Smith-Waterman algorithm. All the tests presented have been conducted on synthetic data for challenging, yet realistic constructs - and on real data gathered during studies on a metabolic engineering example (lycopene production).

## Introduction

### Overview

**FIGURE 1:**
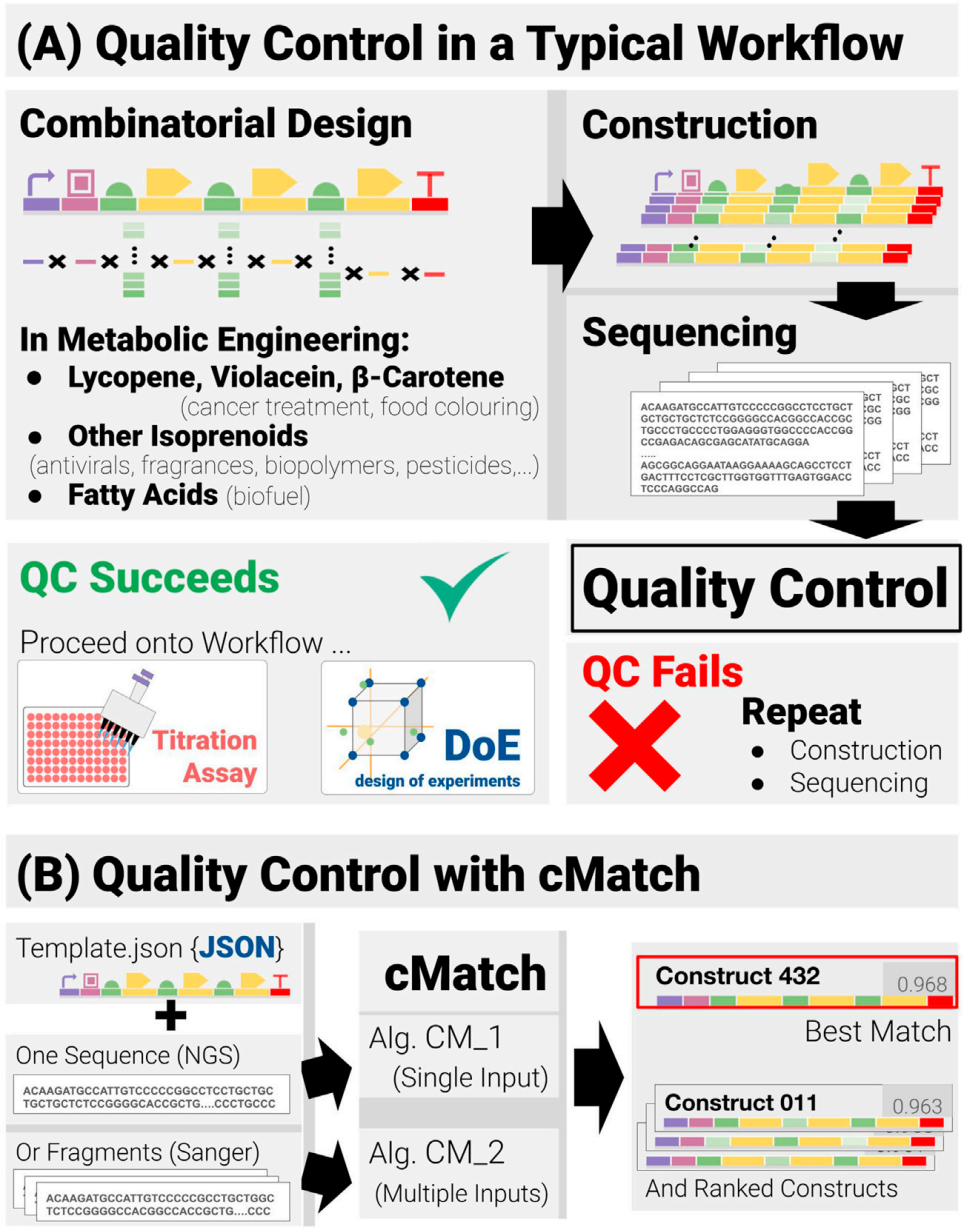
Quality control in combinatorial pathway engineering workflows **(A)** Quality control lies at the junction between the construction and assay phases of the workflow and acts as a binary check on whether the workflow may proceed or if construction and verification must be repeated. **(B)** The quality-control software cMatch uses two types of inputs: a JSON template encoding the positional and combinatorics constraints for a given search space, and sequence data. Sequence data can either be single-input, long-read data (as with next-gen sequencing) or multiple-input, short read data (typical of Sanger sequencing). Two algorithms, CM_1 and CM_2 have been implemented—each tailored for a type of input (CM_1 for single input, CM_2 for multiple input). cMatch returns a best match, as well as a ranking of all the construct in the search space (or an error log).

With the rapidly developing international interest in sustainability and the move away from economic reliance on hydrocarbons, a more bio-based economy requires the development of a range of different biologically based methodologies and processes (Kitney et al., 2019; Bell et al., 2021). For industrial applications these need to possess high levels of reliability and reproducibility. Inherent in this approach is the need to move away from human-based operations to much higher levels of automation, AI and machine learning. As will be described later in the paper, an aspect of metabolic engineering—namely, combinatorial pathway engineering - is seen as an important development area. Combinatorial pathway engineering workflows are iterative, Design-Build-Test-Learn workflows (Carbonell et al., 2018; Hillson et al., 2019; Opgenorth et al., 2019) based on a four-stage process: Combinatorial Design, Construction, Titration Assays and Data Analysis. Between the construction and assays stages, lies a verification phase—Sequencing followed by Quality Control (QC) (Figure 1). QC is crucial from a reliability and reproducibility standpoint. The core subject of this paper is a new methodology called cMatch that makes a significant improvement to QC.

Metabolic engineering (ME), can be defined as the “purposeful modification of intermediary metabolism using recombinant DNA technology” (Cameron and Tong, 1993). It aims to maximise the production of a target metabolite, by modifying native metabolic pathways and/or introducing heterologous pathways, to achieve, in a reliable and reproducible manner, economically viable metabolite yields and production rates. In this context, combinatorial pathway engineering represents a relatively recent and important family of approaches to metabolic engineering. Here, one or more of the pathway components are made to vary simultaneously - in marked contrast to the classic approach, based on identification of bottlenecks in the pathway and subsequent rounds of gradual optimization to lift these bottlenecks (Yadav et al., 2012; Xu et al., 2017).

The development and adoption of combinatorial pathway engineering has been made possible by the convergence of advances in several domains. The first is the availability of efficient DNA modular assembly methods. A range of DNA assembly methods have now been developed (Ellis et al., 2011; Casini et al., 2015) that make extensive use of characterized biological parts and libraries - such as CIDAR Moclo (Iverson et al., 2016) or BASIC (Storch et al., 2015). It is, of course, in general desirable and efficient to use such characterized libraries (Bultelle et al., 2016) and match design with construction. In the context of pathway engineering, these characterized libraries of elements (including plasmid backbones, regulatory elements such as promoters and RBS, CDS, terminators) can be utilized to introduce genetic diversity by offering several levels of gene copy number, plasmid copy numbers, transcription and translation levels, enzymatic activity among others (Jeschek et al., 2017).

Combinatorial pathway engineering is appealing as it yields, with only a few degrees of freedom, a large number of levels over which to tune intricate pathway expression. It also does not require as deep a knowledge of the pathway as the classic debugging approaches (Naseri and Koffas, 2020). But it is not without its own set of challenges. For example, consider the 5-gene violacein pathway (Myeong et al., 2016) arranged in a single operon. A simple translation-based optimization (where RBS in front of each enzyme coding region is changed) yields 10^5^ possible combinations for a small library of 10 RBS. Varying more components in the design (e.g., the promoter driving the operon or adding degradation tags to the enzymes) expands the design space by further orders of magnitude. Such large spaces fast become prohibitively expensive and difficult to investigate by brute force or even one-factor-at-a-time methods (the issues of factor interaction and non-linearity notwithstanding).

The set of methodologies known as Design of Experiments (DoE) (Gilman, 2021) have become popular to deal with the design and highly-complex multifactorial optimization of large-scale combinatorial spaces. DoE methods are very amenable to automation, as works by (Carbonell et al., 2018) and (Rajakumar et al., 2019) have demonstrated. They have been adapted to identify the media and culture conditions that maximize yield of the metabolic pathway (Singleton et al., 2019) (Azubuike et al., 2020), or the optimization of a cell-free system (Spice et al., 2020). In the context of metabolic pathways, these methodologies have been applied to choose regulatory elements such as promoters (Blazeck et al., 2012) and 5′UTR (Salis et al., 2009), or to modify other popular dosage parameters such as plasmid copy number (Ajikumar et al., 2010) and codon usage, (Hanson and Coller, 2018; Wu et al., 2019). (Xu et al., 2017) showed how the five gene violacein pathway could be simplified to three operons as two of then encoded enzymes had no impact on the output. DoE methods can be unsuited to the estimation of parameters. Optimal Experimental Design (OED) can be used when a model is available for the pathway. The goal then becomes to design a set of experiments so the model parameters may be estimated more reliably (Abt et al., 2018; Smucker et al., 2018). OED typically aims to maximize the information content (computed from the Fisher Information Matrix or with some Bayesian approaches) of the new experimental iteration (Nishii, 1993). Despite their proven track record in reducing the number of experiments and improving reliability, both sets of methods still require sampling at least a few percent of the design space (Lee et al., 2013) - potentially a very large number.

### Requirements for Quality Control in Pathway Engineering

QC takes place post construction, transformation and growth. It has a simple goal, verifying that the genetic material contained in a cell matches what was intended to be built. In terms of reproducibility/reliability, the QC step is crucial. Failure at this step requires repetition of the construction and sequencing steps, while it should only be possible to proceed to the next stages of the workflow once the QC standards have been met.

The development of cMatch, which is the new methodology at the core of this paper, was born out of the practical experience in the Kitney Lab gathered during a succession of projects in pathway engineering (Exley et al., 2019) and automated robotic platforms (Reynolds et al., 2017; Suckling et al., 2019). These projects identified the need to make QC a frictionless step in the workflow, capable to reliably, and robustly, perform at scale and speed. It was also decided that QC tools should be accessible to everyone, and in particular, that it should not take dedicated hardware (no GPUs, no high performance clusters) to run them. Finally, sequence analysis for a typical construct should take less than a few minutes - our strategy for removing the bottleneck. cMatch has been specifically developed to exploit and tackle a set of five features and constraints that are typical of large-scale combinatorial spaces and metabolic engineering. These are as follows.

#### Tackling the Problem Requires Automation and Programmatic Access

Quality control quickly becomes a bottleneck when too many sequences need to be verified - as is the case with large design spaces. The standard way of identifying a genetic constructs and its different components involves loading the results of a Sanger sequencing onto a DNA editing software, e.g., Benchling (https://www.benchling.com), and painstakingly searching for the possible components one by one. This quasi-manual process is extremely time consuming (up to several hours for a few sequences) and error prone. It also scales very poorly with the number of constructs and with the complexity of the constructs. Differentially identifying close constructs (e.g., using closest RBS) is extremely challenging. The process also becomes vastly more cumbersome when comparison is made against not one, but several members of the same design space. Finally, there is also no way to assess the reliability of the process—except by repeating it.

To tackle QC at scale, this process needs to be automated. Decision criteria (including for failure or success) also need to be built into the process. All parameters driving the process should be transparent and accessible to the user to change. Finally, to make results comparable, the process needs to rely on, and return objective quantitative metrics.

#### The Synthetic Constructs Share a Well-Defined Structure, Thanks to Their Modular Design

Modularity has been one of the main drivers of the development of synthetic biology, alongside characterization and standardization (Kitney, 2009). These principles have facilitated the creation of complex systems from the combination of well-understood, standardized components in disciplines such as electrical engineering, or computer science.

(Hartwell et al., 1999) argued for the recognition of the concept of functional modularity as a critical level of biological organization. Modular design, which aims to combine such functional modules, has been the main driver behind the development of libraries of standard characterized components - from simple regulatory elements such as promoters.

(Alper et al., 2005; De Mey et al., 2007; Redden and Alper, 2015; Zucca et al., 2015; Rao et al., 2021), or degradation tags (Cameron and Collins, 2014) to more complex components such as logic gates (Nielsen et al., 2016). Large repositories of DNA parts, such as the iGEM Registry of Standard Biological parts (https://parts.igem.org), JBEI-ICE (Ham et al., 2012)), Addgene (Kamens, 2015) or SynbioHub (McLaughlin et al., 2018), are now routinely used to assist the engineering of synthetic DNA circuits and plasmids (Timmons and Densmore, 2020).

Synthetic constructs generated from a modular design share very specific features:

- The type and relative order of their components are known.
- Their components are drawn from known lists of elements (often from well-characterized libraries)
- The interfacing of their components is also well-defined. Most designs will use non-overlapping components; when this is not the case the overlaps can be strictly defined as they are parts of the design

#### A High Level of Precision Is Required in the Identification of Some Components

The biological functions encoded in the constructs have sequences spanning several orders of magnitude (CDS are one kb or more, promoters around a 100bp, UTRs around 50 bp or less, while degradation tags are often less than 10bp long). Some very short regulatory components such as RBS can be rationally designed to span specified ranges of strength (Jeschek et al., 2016), but minor changes in their sequence will have a significant effect on their strengths (Salis et al., 2009). These components must therefore be identified (sequence and location) with the utmost precision.

#### A High Level of Precision Is Also Required for Components Interfaces

Context is the set of interrelated factors that modulate the operation of biological processes—these factors are either composition-specific, environment-specific or host-specific (Cardinale and Arkin, 2012). Environment and host-specificity are irrelevant to the problem addressed here. Composition specificity is crucial on the other hand, and takes many forms. For instance, in an operon, gene order influences effective transcription rates (Nishizaki et al., 2007). Constructs are also at risk of several forms of genetic instability, most commonly deletions caused by homologous recombination and indels—risks that can be estimated with the EFM calculator (Jack et al., 2015). Of all composition factors, the interfacing of adjacent regions is the most important for QC. Expression of functional components is indeed affected by short adjacent sequences (Espah Borujeni et al., 2014; Taylor et al., 2019; Tietze and Lale, 2021). A calculator such as the RBS calculator (Salis et al., 2009) (https://www.denovodna.com/) now requires 35 bp upstream and 60 downstream to estimate the translation rate of an RBS.

The greatest care therefore also needs to be paid to the accurate identification of the regions adjacent to the regulatory elements, as well as to the regulatory regions themselves.

#### Sequencing Data Will Be Short-but Possibly Spread Over Several Sequences

Typical constructs will only include a small number of genes (less than 20, often far fewer as pathways will be refactored over several plasmids if they are too long). Sequences to analyze will therefore be several kbp-long or less (considerably less than the length of genomes). Practically, this implies that cMatch can rely on one of the most precise matching algorithms, instead of a popular algorithm such as BLAST (Altschul et al., 1990) which is better suited to genome length sequences. Finally, sequencing data for a construct will consist of one sequence spanning the whole construct, or several shorter sequences covering complementary fragments of the construct. Discussion in this paper will be mostly based on the use of Sanger sequencing (Sanger and Coulson, 1975; Sanger et al., 1977). Sanger sequencing is the founding method in DNA sequencing, but it remains popular for applications where high throughput is not needed, thanks to its wide availability from a range of for-profit companies. Sanger sequencing is affected by poor quality in the first 50 bases of the sequence due to primer binding and deteriorating quality after 700–900 bases. Quality of the sequence can be estimated with a base calling software such as Phred (Ledergerber and Dessimoz, 2011). Although cMatch was not tested on data generated by more recent methods (Shendure et al., 2017) such as Next-generation sequencing (NGS) (Burgess, 2018; Slatko et al., 2018), which yield much longer reads with lower error-rate, the authors have no reason to doubt the software will behave any differently to the way it behaved with Sanger data.

### Construct-Matching with cMatch

**FIGURE 2:**
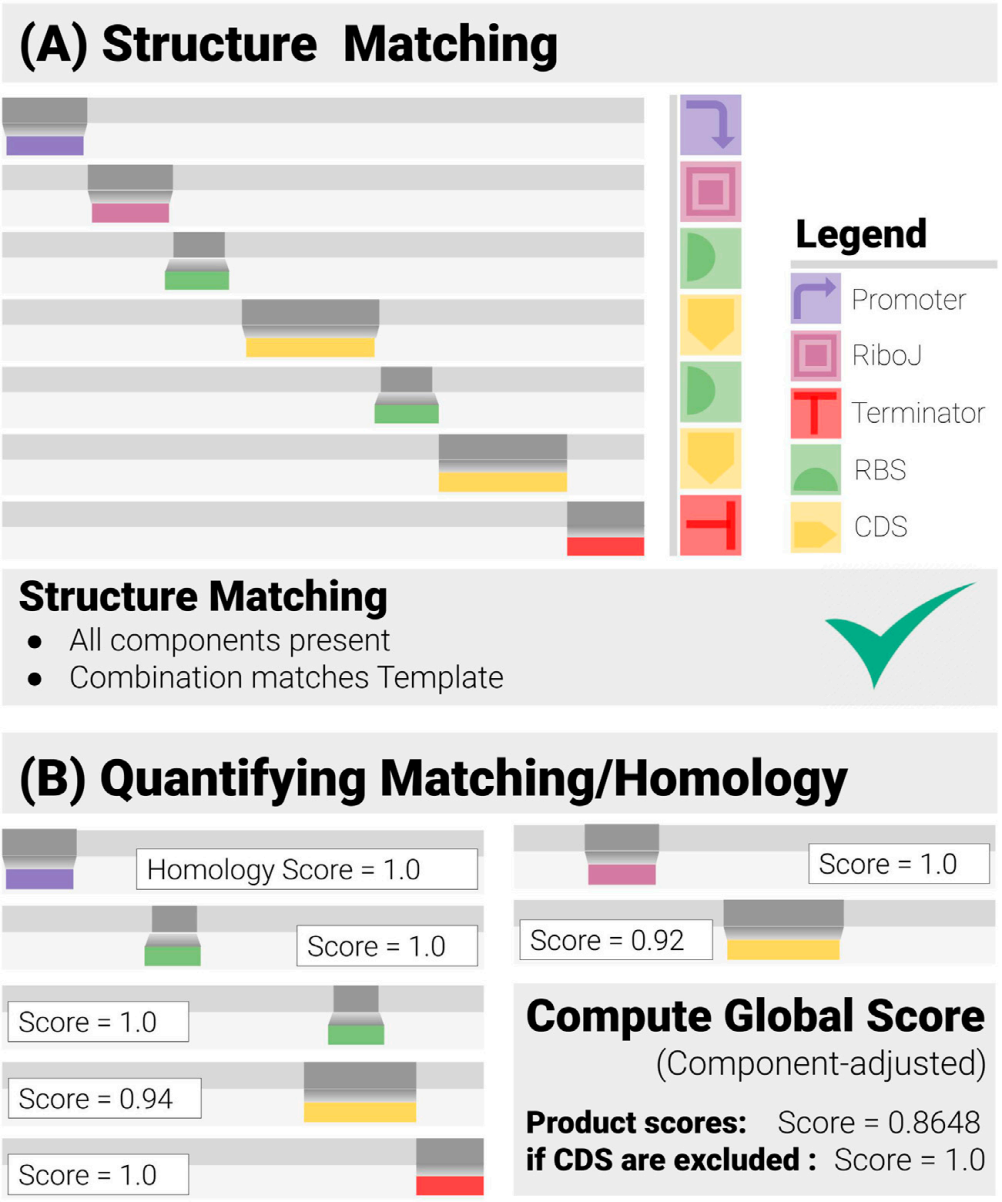
Construct Matching in Action (for a Two-gene Operon Design). Construct-matching performs two complementary tasks. **(A)** Structure Matching: this step and checks whether the data correspond to an admissible construct, that is contains all the specified components in a combination that matches the design template. **(B)** Quantification of the Matching: each component has its own matching/homology score, from which a global score is derived.

To exploit the structures of the constructs, cMatch operates at the functional (component) level. Since synthetic designs encode a set of biological functions, and add a set of positional constraints, cMatch searches for components encoding given functions, and checks if their combination is admissible (Figure 2A). We call this operation “construct-matching.” It is a more thorough operation than simple sequence-matching or alignment, as it aims to answer a set of questions regarding the modular structure of the construct, as well as how well each component matches the data, including

● Structure matching: Does the construct match the design?
○ Are all intended components present ?
○ Does their order match the construct design ?
● Quality of the matching: How close is the matching?
○ Are there any components affected by Mutations? If so, to what extent
○ Are there any insertions and/or deletions ?

cMatch derives a standardized matching score for each component. These scores and their weighted average are then used to automate the decision-making and quantify the reliability of the prediction through a set of global matching scores (Figure 2B).

Separating the components in the construct helps address several of the previously listed challenges.

- All functional modules are dealt with in parallel. Homology (matching) scores can be standardized against their lengths, yielding an immediate insight into where the discrepancies between data and ideal sequences are located. This is important when dealing with short, regulatory components, and their adjacent regions.
- It is easy to generate global matching metrics from the individual component scores. Equal weight can be given to the components and the geometric mean can be calculated. Conversely, some components, such as CDS, may be omitted from the global scores if the user decides mutations in these regions are of little concern.

cMatch is built on top of the highly validated Smith-Waterman algorithm (Needleman and Wunsch, 1970; Smith and Waterman, 1981). This choice was born out of the need for a high level of precision in the identification of some components and their interfaces. These stringent requirements place cMatch in sharp contrast to applications such as barcoding (Woodruff et al., 2017), where short barcode sequences (with a space of possibilities in the billions) are used to identify constructs in a design space of several thousands/millions. Exact string comparison, as implemented in annotation tools such as Benchling’s Auto Annotate (https://help.benchling.com/en/articles/2835801-auto-annotate-sequences), is the fastest form of matching but it is impractical for QC, as it does not offer any way to deal with mutations or indels (insertions/deletions), and more generally fails as soon as data differ from the expected sequences (without being able to assess to what degree they differ). The two standard algorithms in bioinformatics Smith-Waterman and BLAST (Altschul et al., 1990) were considered for cMatch. BLAST is very popular thanks to its speed and is the algorithm of choice to deal with long and genome-length sequences (Goujon et al., 2010; Donkor et al., 2014) stored in the many biological databases (Lakshmi and Ramyachitra, 2020). But it is not as precise as Smith-Waterman, which is designed to return the optimal local alignment (Wieds, 2007). Smith-Waterman is much slower and is costly in terms of computer power and resources. This concern was overlooked, as the sequences to be analyzed and the corresponding constructs are several kbp-long or less (considerably less than the length of genomes), which remains tractable. The fact the algorithm is deterministic (unlike BLAST), which is important for reproducibility, and the availability of a highly validated biopython implementation (Chapman and Chang, 2000) were compounding factors for our choice.

It is worth emphasizing that cMatch is not a sequence annotation tool (although its matching phase has a lot in common with such tools), but a QC tool. Indeed cMatch relies on knowledge of the structure of the (ideal) construct - from which an annotation can already be derived. Some sequence annotation (e.g., including a comparison of sequencing data to the ideal sequence) is a possible by-product of the construct-matching process, but not its main objective.

Comparisons with the recent range of auto-annotation tools for plasmids and constructs of similar length remain instructive. Beside its choice of sequence-matching method, the component-matching phase of cMatch differs from tools such as Benchling Auto-annotate (https://help.benchling.com/en/articles/2835801-auto-annotate-sequences) and pLannotate (McGuffie and Barrick, 2021) in several ways. Unlike pLannotate, it does not use external databases as sources for the components. CMatch also offers full programmatic access and lets the user change the matching parameters. Only the sequence-annotate features offered by the recent SYNBICT (Synthetic Biology Curation Tools https://github.com/SD2E/SYNBICT) project offer a similar level of flexibility (and also use pairwise matching).

But where cMatch differs from this set of tools in an important way is at the second stage: it goes much further than sequence annotation, as it performs a set of QC operations on top of component matching, and offers ways to quantify the entire process (only SYNBICT defines a threshold to accept/reject annotations, but it does not utilize a template in any way). cMatch also exploits modularity in order to offer comparisons not against one construct, but an entire design space.

## Methods—Algorithms for Construct-Matching

We now present our solution to the construct-matching problem. A lightweight data structure encoding a construct template and the search space, is introduced in *Supporting Data Structures* section. Two construct-matching algorithms are presented. The first algorithm is a sequence-based algorithm based on a brute force investigation of the design space (*Sequence Matching Algorithm* section) - it is included for comparison purposes and to highlight the benefits of the component-matching approach adopted by the other algorithms. Finally, the construct-matching algorithm (*Component-Matching Algorithm* section), based on a component matching strategy and step-by-step reconstruction is presented, as well as an extension to deal with multiple inputs.

### Supporting Data Structures

Construct-matching has two distinct components:

● A construct to be identified (the “target” of the analysis) by way of sequencing results. All sequences take value in the IUPAC dictionary (https://www.bioinformatics.org/sms/iupac.html). These sequences are the input of the algorithm.
● A “search space.” The constructs contained in the search space will be referred to as “candidates” - they are possible output values for the identity of the target construct. The search space is left to the user to define. In a straightforward QC scenario (henceforth referred to as “minimal QC,” the search space only includes one construct - the construct intended for construction. In more thorough QC checks, it can extend to the whole design space for the construct (we call this scenario “maximaml QC.” The search space is specified by two search constraints (they are the parameters of the algorithm)
○ A template describing the modular structure of the construct (type of the components and relative order) and thus providing a set of structural constraints for the space of possibilities
○ For each component of the template, a library of admissible elements—providing a description of the combinatorial element of the problem

**FIGURE 3:**
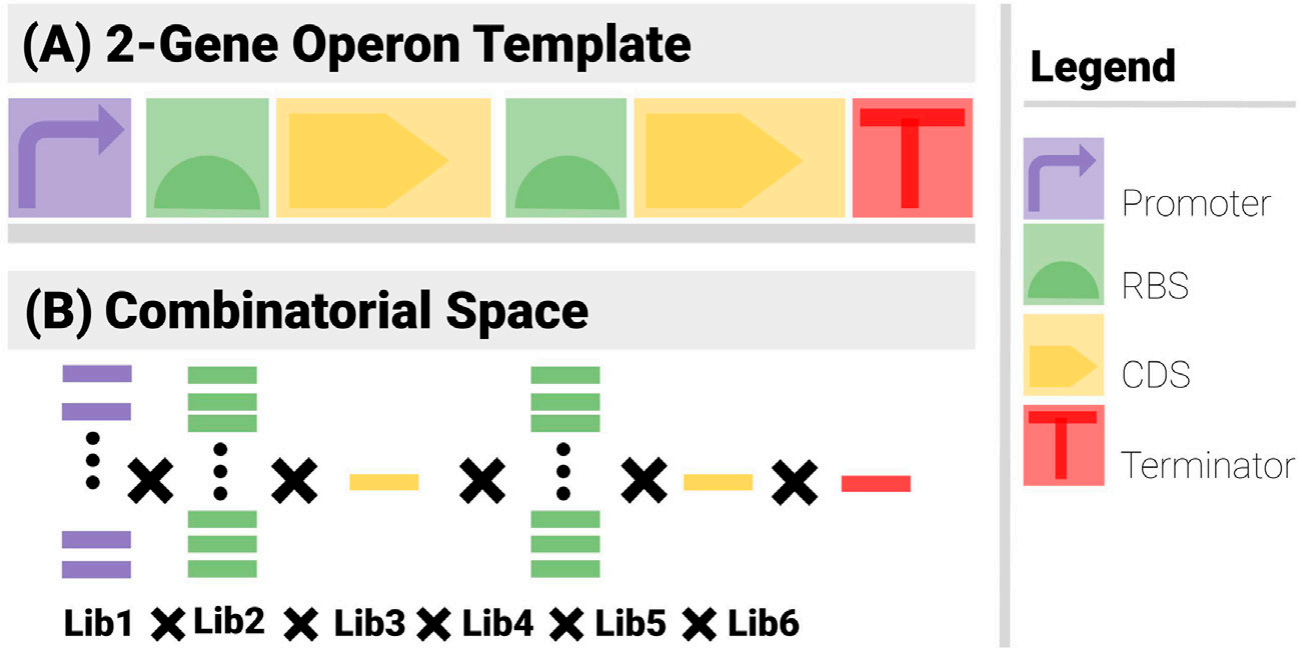
The two sets of search constraints for a 2-gene operon. **(A)** The template specifies the types of the components and their positional arrangements. **(B)** The list of possible elements for each component yields the combinatorial element of the problem.

Figure 3 shows the search constraints for a two-gene construct example.

● Template (Figure 3A): The construct has 6 components (1 promoter, 2 RBS, 2 CDS and a terminator) arranged in an operon pattern.
● Combinatorics (Figure 3B): A promoter library of 5, 2 RBS libraries of 10, 2 CDS libraries of 1 and a Terminator library of 1 are used.

Even with such a simple example, the power of the template is apparent. There are 5 × 10 × 1 × 10 × 1 × 1 = 500 admissible combinations. If the relative order of the components is not imposed, there are 6! = 720 as many ways to arrange the components, i.e., 360,000 (mostly non-functional). These numbers further increase exponentially if components can go missing or feature more often than once.

CMatch requires knowledge of component location and interfacing for the template description. Component location could either be absolute or relative, but absolute locations were rejected as impractical. Sanger sequencing indeed often returns sequences of poor quality at the start and end (possibly truncated, often with bases with a phred-quality score too low to have a value safely assigned to them). Since sequence data are 1D structure (strings), it is enough for components to be listed in relative order, in the 5′ to the 3′ direction. The template structure encodes this order explicitly, through the value of “template_location” - an integer starting at 1 (to be more user friendly) and incrementing as one moves 3′-ward. This is needed to implement the slicing operations necessary to combine reconstructions when several sequences are used as inputs. The template also specifies a global parameter for component interfacing: a maximum overlapping term epsilon (in bp, default value set to 0) to deal with scars between components, deal with undefined components, and more generally provide flexibility when data are poor quality.

For simplicity reasons, cMatch encodes both template and combinatorics constraints, as well as sourcing information in a single JSON file (see Supplementary Material for examples of valid JSON files).

The “template” portion of the file encodes the construct template (structural constraints and corresponding combinatorics). A template lists its components, while a component lists the source for the library containing the sequence data, as well as positional information for the component. In the case of the two-gene operon used as an example in Figure 3A, the “template” structure is as follows. To make the hierarchical structure more evident, the instances of components have been shaded in green.


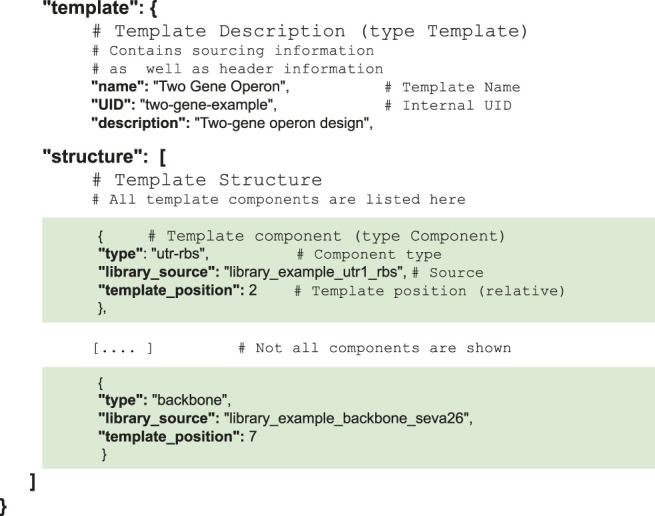


Sourcing information is encoded into another portion of the file (in a structure called “component_sources”). All component libraries are listed—each one points to a repository address, and lists its elements. An element points towards the file containing the sequence of the element. To simplify data import, only text, JSON, GenBank and SBOL file formats are supported. In the case of the two-gene operon, the “component_sources” structure is as follows. To make the hierarchical structure more evident, the instances of libraries have been shaded in blue.


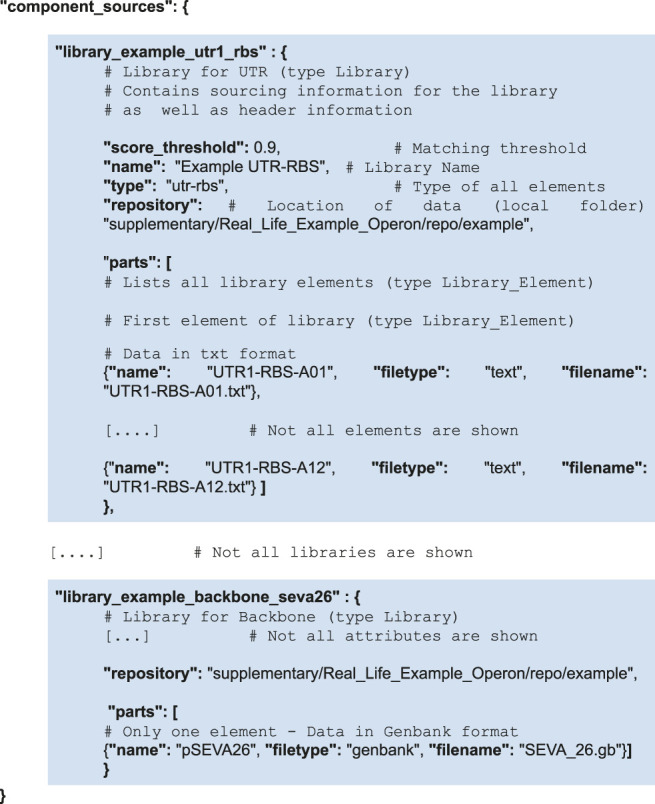


### Sequence Matching Algorithm

**FIGURE 4:**
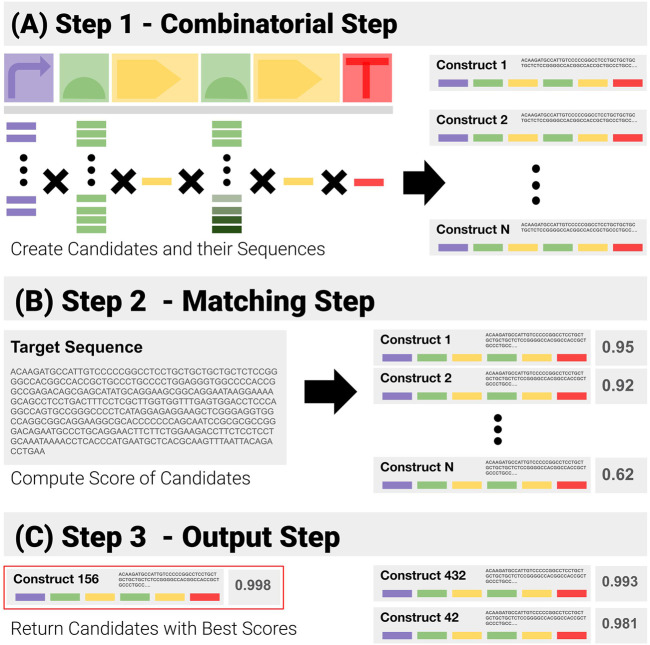
Visual Representation of the Construct-Matching Algorithm CM_0. CM_0 is a sequence matching algorithm that fails to exploit modularity. It is made of three steps. **(A)** Step 1 - CM_0 generates the sequences for all possible constructs in the search space. **(B)** Step 2 - These candidate sequences are matched to the input sequence—returning for each a global matching score. **(C)** Step 3 - Constructs with the highest scores are returned.

Given prior knowledge of a construct template, and its libraries of components, a simple solution to QC consists of a purely combinatorial generation of all the candidates, followed by brute force matching of their sequences (Figure 4):

1) Combinatorial Step (Figure 4A): generate all possible combinations in the search space from the template and libraries of components, and the corresponding sequences.
2) Matching Step (Figure 4B: for each generated sequence, compute the matching score to the target sequence with the Smith and Waterman pairwise alignment algorithm.
3) Output Step (Figure 4C: return the combination(s) with the highest scores.

Such an approach does not seek to exploit the modularity of the constructs. Issues with such an approach are four-fold.

1) It is very rigid in the way it builds the candidate sequences, and makes it difficult to apply overlapping options.
2) The computational cost of a pure combinatorial approach fast becomes prohibitive, as the number of candidate constructs increases exponentially.
3) The performance of pairwise matching algorithms degrades fast as the sequences increase in length - due in no small part to their attempt at finding the best matches by inserting and deleting base pairs (see benchmark results in *Results - Applications and Benchmarks* section). Matching an input sequence against complete construct sequences will quickly prove costly in terms of time and memory utilization.
4) The approach naturally gives more weight to the longer components like the CDS to the detriment of very short, yet important ones like RBSs, because it only computes a global matching/homology metric.

In the rest of this work, this algorithm is called *CM_0* (“Construct Matching 0”) - the null index indicating that it is a stepping stone to the final algorithm(s). All construct-matching algorithms are tested against CM_0 in the Results section.

### Component-Matching Algorithm

**FIGURE 5:**
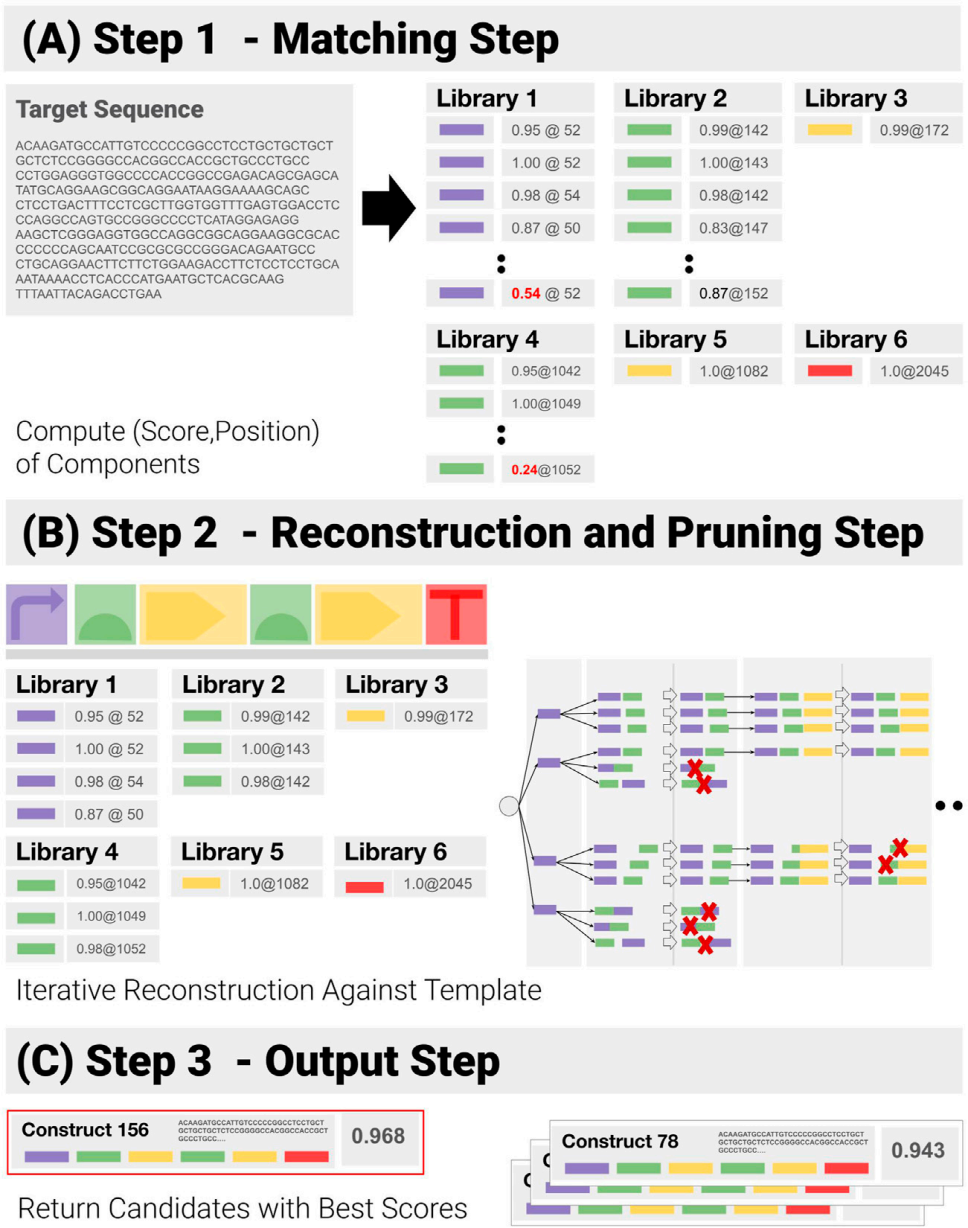
Successive Steps of the Construct-Matching Algorithm CM_1. **(A)** Step 1 - CM_1 first looks for all components in the target sequence. Poor matches (low score, in red) are pruned out at this early stage. **(B)** Step 2 - Components identified in Step 1 are recombined into a list of all admissible constructs. Dynamic reconstruction was implemented. A matching score is generated from the individual component matching scores for all possible constructs. **(C)** Step 3 - Constructs with the highest scores are returned.

We have developed a component-based algorithm (referred to as *CM_1*), which performs the operation we have called construct-matching. Unlike CM_0, CM_1 utilizes the information contained in the template, and focuses on function-encoding components and their admissible combinations - before ranking them. A pseudo-code implementation of CM_1 can be found in the Supplementary Section S3. CM_1 proceeds as follows (Figure 5):

1) Component Matching Step (Figure 5A): This step looks for the components in the target—looping over all libraries of components, to match each library element to a subsequence of the input sequence, and assigning a matching score (normalized by the length of the component) and a position to each of them in the process. Pruning follows: only matches with a score above a user-specified threshold are kept.
2) Reconstruction and Pruning Step (Figure 5B): Identified components are recombined into a list of admissible constructs. Rather than using a purely combinatorial approach, where all possible combinations are generated and then pruned against the template, an iterative reconstruction—based on dynamic programming has been implemented (See Supplementary Section S2). A combinatorial approach proves extremely costly when the input contains repetitions and components are detected in several locations, as the number of possible combinations expands by several orders of magnitude (See *Synthetic Benchmarks* section for an example). Instead, the reconstruction applies positional constraints (E.G. “component 3 must be located before component 4”) as early as possible to prune out entire branches of the reconstruction tree.
3) Output Step (Figure 5C): At the end of the reconstruction, all remaining paths are assigned a global score (we use the geometric mean of the components scores). The combinations with the highest scores are returned.

By performing construct-matching rather than mere sequence-matching, CM_1 returns a great deal more information than CM_0 - it identifies and locates all the components in the construct, confirms their combination matches the template, and returns individual homology/matching scores for each of these components - a crucial source of information to identify where the mutations are. Having access to a lower level of information allows us to deal with the function-encoding components (however long or small) in parallel. This allows us to apply options to specify acceptable interfaces between these components, which is useful when data are of poor quality and components can not be precisely located (see the lycopene example, in Results *Real Life Example - Lycopene Operon* section). It also allows us to generate global matching metrics from the individual component scores.

Working at component level opens up a set of strategies to limit computation times and resource utilization. First it avoids matching long sequences to long sequences and can be parallelized. Also, it enables us to avoid combinatorial explosion, by only using the best matches from each library, and then pruning reconstruction paths as early as possible.

CM_1 relies on a sequence being available that covers the whole construct, and that can therefore be used as an input. Such an assumption may not be met in general use. Using multiple sequences as inputs instead of a single one is very common with Sanger sequencing, as it only returns reads of a limited length (∼500–700bp). Using several sequences also gives flexibility regarding the portion of the construct that requires identifying. Such flexibility is desirable, as some components are more important than others for given applications, and their presence can be deduced thanks to prior QC checks. In the practical application described in *Real Life Example - Lycopene Operon* section, reverse primers originating in the CDS were used that only covered a few hundred base pairs of the CDS - but gels were run to check the constructs were of the expected sizes.

To widen the application range of the construct-matching algorithm, we have extended CM_1 so it may support multiple sequences as inputs. The new algorithm (henceforth referred to as CM_2) utilizes a split-apply-combine strategy (Wickham, 2011), where the first step of the process (the split phase) mirrors the sequencing of fragments of the construct. CM_2 proceeds as follows:

1) Partition Step: From the input data (Sub_sequence_i_) and the global template, a set of sub-problems {(Sub_sequence_i_, Sub_Template_i_)}_i=1 … N_. are generated.
2) SubSequence Analysis: CM_1 is applied in parallel to all the sub-problems - which are also all cheaper to solve than the original problem due to their smaller size.
3) Recombination Step: The results of the individual analyzes are combined into a final construct matching the original template

CM_2 implements a pair of simple operations on the template structure, namely slicing for the partition step and addition for the recombination step. Both operations are detailed in the Supplementary Section S4. Instructions regarding the slicing are contained in the JSON input—instructions used in the lycopene example are as follows:


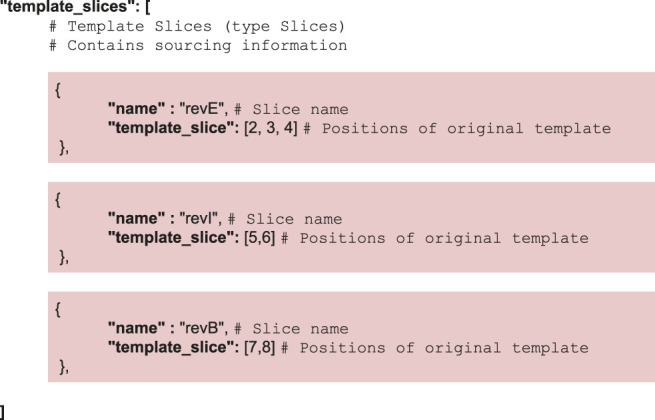


## Results—Applications and Benchmarks

The performance for all three algorithms presented in *Methods—Algorithms for Construct-Matching* section will now be compared. All benchmark results are derived for a series of realistic scenarios based on common construct designs. The chosen pathways (both for naturally-occurring pigments) are classic examples in combinatorial pathway engineering. To test whether the construct-matching algorithms conform to our original remit regarding speed and common hardware, all computations have been performed on the personal daily driver of one of the authors, a Lenovo ThinkPad X220.

Finally, in these benchmarks two forms of quality control - of opposite complexity will be run:

- Minimal QC: data obtained from sequencing of a construct are compared to the construct itself. This is the most common use of quality control—performed to check that what was actually built matches what was intended.
- Maximal QC: the data are compared to the entire design space of the construct. This is a more thorough form of QC, as it offers a form of insurance against errors such as mislabelling, and further tools to estimate the overall reliability of the construct-matching process (see Discussion). Such extensive search is also needed in other applications such as the pooled approach study discussed in *Discussion* section

### Synthetic Benchmarks

**FIGURE 6:**
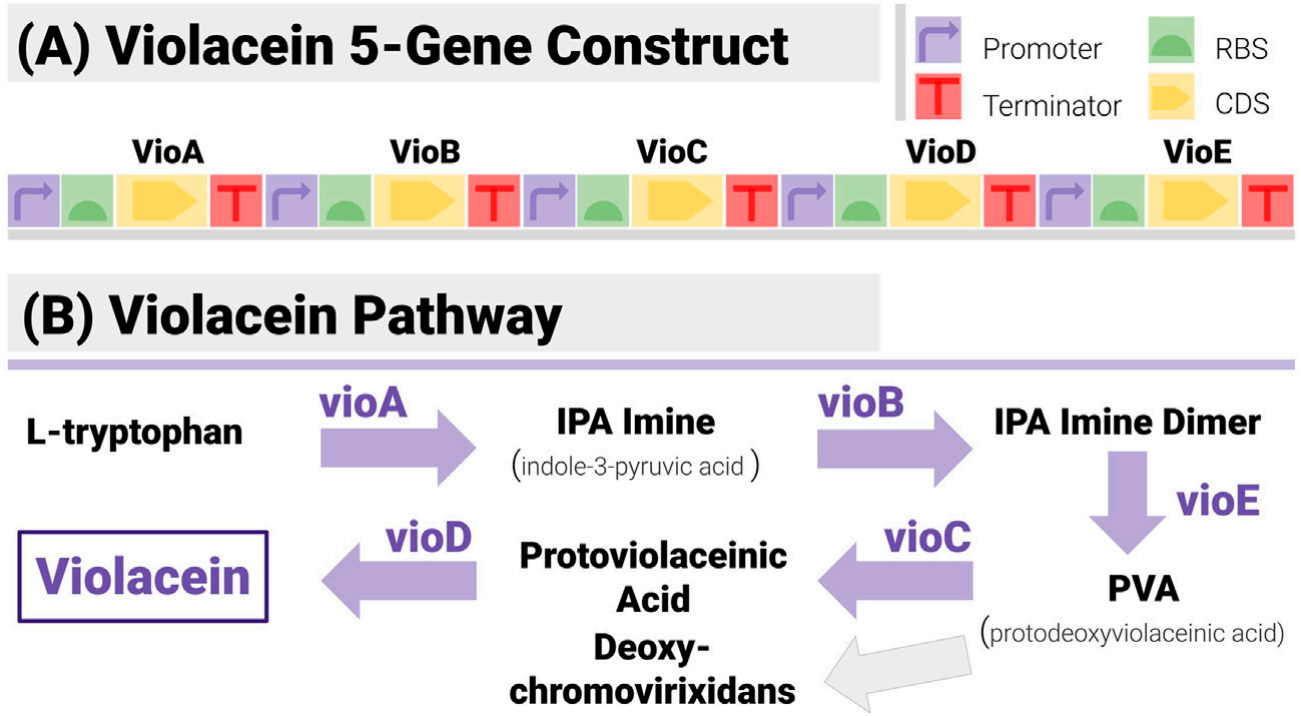
The 5-Gene Violacein Pathway and its Implementation with a TU-Design. **(A)** The design is made up of 5 transcription units (hence 20 components) in a set gene order. All units share the same promoter (BBa_J23101) and terminator (BBa_B0015) - providing a stiff test for the algorithm, due to the large number of potential repetitions. **(B)** The violacein pathway is made up of five enzymes (VioA to VioE). Its associated regulatory mechanisms have been characterized in several strains including *E. coli* and Yeast.

The construct matching algorithms were first tested on synthetic data for a construct (Figure 6A) encoding all five enzymes in the violacein pathway (Figure 6B). Violacein is a classic test bed since the genes encoding the enzymes necessary for its production, and the associated regulatory mechanisms have been characterized in several strains (Myeong et al., 2016). It is also a metabolite with notable industrial applications whether as a dye (thanks to its vivid purple colour) or for medical applications (Durán et al., 2007).

Construct design was based on a simple TU (transcription unit) pattern (Figure 6A):

- Each enzyme is encoded with its own TU. TUs share the same promoter (BBa_J23101) and terminator (BBa_B0015), both mainstays of the iGEM Registry.
- The design fixes the gene order to VioA-VioB-VioC-VioD-VioE.
- All UTRs can be varied, as per (Salis, 2009; Blazek, 2012). All positions use the same library of 5 RBS from iGEM registry (BBa_B0030 to BBa_B0033 and BBa_B0064, chosen to only differ by a few base pairs).

With this design, the constructs are made of 20 non-overlapping components, and the design space is of size 5^5^ = 3,125. The TU-based pattern is a challenging case for a construct-matching algorithm, due to the large number of repetitions in different locations (all constructs have 5 identical promoters, and 5 identical terminators). RBS libraries have been chosen to make reconstruction as difficult as possible (they are identical).

All input data for the violacein example are synthetic data—generated from the construct template and the specified components libraries. This was done to be able to compare the output of the algorithms with the ground truths (and make sure the correct constructs were identified). All the logs and output files for all the tests that are discussed below can be found on the cmatch Github repository at https://github.com/kitneylab/cmatch. Listed times are indicative of the performance of the various algorithms, but will vary depending on the user’s machine.

#### Testing Algorithm CM_0

The predicted limitations of CM_0 can be illustrated with a few tests on the synthetic data.

The first test with CM_0 was a minimal QC scenario; the construct Vio-0000 was used (a construct with 5 identical RBS B0030), and the operation was repeated N = 10 times. The average run time was 36.60 ± 0.73 s. When 10 different constructs (Vio-0000 and 9 other constructs drawn at random) were used as targets and matched against themselves, the average running time was 37.89 ± 2.26 s (N = 10 repeats for each target). In all cases, a perfect score of 1.0 was returned every time—confirming that the Smith-Waterman algorithm correctly matches identical sequences. Inspection of the results show the times were distributed along a bimodal distribution (previous results were unimodal) with a first mode around 36 s and a second mode around 42 s. No link between the input and belonging to a mode was apparent. Those results were attributed to resource limitations in the system. This stress was expected, as Smith-Waterman is resource-intensive and the target sequences were almost 8500bp long.

Another predictable limitation of CM_0 is apparent when the search space is expanded - from a few constructs of interest to the entire design space (maximal QC). As CM_0 is expected to perform linearly with the number of candidates, identification was first performed with one target and 10 candidates (Vio-0000 and 9 other candidates drawn at random) and repeated 10 times. The run time was 370.72 ± 3.99 s. The operation was then repeated with 100 candidates (Vio-0000 and 99 other candidates drawn at random). The test was only run three times (previous results showing little variation), yielding a run time between 56 and 58 min. Extrapolating from these, if the whole design space was used as search space (maximal QC, against more than 3,000 constructs), it would take more than 30 h to identify a target construct with CM_0 - a wholly impractical time.

Finally, because of its global sequence-matching strategy, CM_0 offers few insights into the quality of the prediction. In all instances, the correct construct was identified with a perfect score of 1.0, but several candidates returned scores above 0.99 since they varied only by a few base pairs from the target. Furthermore, a global match does not help locate the discrepancies between data and construct sequences and their significance.

#### Testing Algorithm CM_1

CM_1 has two limiting steps: its matching step (affected by the length of the target sequences, the number and length of the elements in the component libraries), and its reconstruction step (affected by the repetitions in the construct and the matching threshold).

To test the reconstruction phase of the algorithm, tests were run with two threshold levels (0.99 and 0.75), and for two targets of opposite complexity:

- Vio-0007- a construct with 5 different RBS (B0030-B0031-B0032-B0033-B0064)
- Vio-0000 - a construct with 5 identical RBS (all B0030)

Vio-0007 is the easiest case for reconstruction: after the matching step, all promoters and terminators will have been identified in 5 possible locations, but its UTRs will have been identified at a single location. The reconstruction step needs to prune a space of possibility of size 5^10^ i.e., 10 millions (more if the detection threshold is low) down to one construct. Vio-0000 is the most challenging case. After the matching step, its UTRs will also have been identified at 5 possible locations. The reconstruction step needs to prune a space of possibility of size 5^15^ (more than 30 billions). Brute force pruning quickly becomes intractable. The step-by-step reconstruction was developed for such cases, and to prune combinations as soon as they break the template constraints. Performance of the pruning algorithm is illustrated in Supplementary Section S2 of the Supplementary Information on the most computationally-challenging case of maximal QC with Vio-0000.

To assess the influence of the number of elements in libraries—tests were run for the opposite cases of minimal QC (against the target itself) and maximal QC (against all the 3,125 members of the design space). All tests were repeated 10 times. In all cases, the overlapping parameter epsilon was set to 0. Results for all tests are displayed in Table 1.

**TABLE 1:** Performance of the CM_1 Algorithm. Performance was assessed for constructs of opposite complexity and different reconstruction thresholds. Results in the top part of the table for the minimal QC scenario show computation times do not depend on the constructs and threshold. Results in the bottom part of the table for the maximal QC scenario show similar results.

Minimal quality control with CM_1		
	Threshold = 0.99	Threshold = 0.75
Vio-0000	78.70 ± 6.61 s	83.07 ± 7.26 s
Vio-0007	78.80 ± 0.35 s	79.12 ± 1.05 s
**Maximal quality control with CM_1**		
	**Threshold = 0.99**	**Threshold = 0.75**
Vio-0000	109.86 ± 15.10 s	99.41 ± 14.31 s
Vio-0007	104.53 ± 4.47 s	106.43 ± 10.60 s

In the minimal QC case, with both thresholds and both targets, the reconstruction yielded only one combination (the target itself) with a perfect score of 1.0. Overall running time was larger with CM_1 than CM_0, and was independent of the target construct. The run time did not change against the threshold (as there was only one candidate for identification). Split times (see logs) show that identification of the individual components only took a few seconds each (except the longer VioB that took 11 s).

In the maximal QC case, the reconstruction yielded (for both targets) only one possible reconstruction, the target itself, and did so with a perfect score of 1.0 for the higher threshold. With the lower threshold, 2 UTRs passed the matching step for each template position., and the reconstruction yielded 32 possible reconstructions. Final selection then identified the correct construct (the target itself) with a perfect score of 1.0 among all the candidates. As expected, matching against the whole design space took longer than against one construct - between 100 and 110 s. The difference corresponded to the time required to match all the components needed for a full comparison with the design space.

Split times (see logs) confirm that, for both minimal and maximal QC tests, reconstruction times were in the hundredths of a second and the component-matching step was the limiting step of the whole process. Variations between runs showed similar results as for CM_0 - times were either clustered around a main value close or spread in a tail. As with CM_0, these results were attributed to resource limitations in the system.

#### Testing Algorithm CM_2

CM_2 has the same limiting factors as CM_1. All tests in this section were therefore identical to the tests run on CM_1: same targets [Vio-0007 and Vio-0000, same two threshold levels (0.99 and 0.75), and overlap epsilon = 0; All tests were also run 10 times] for a minimal QC scenario and a maximal QC scenario. Input sequences were generated for the five separate transcription units in the construct (covering them in their entirety).

Results in both tests (Table 2) show CM_2 performed faster than for CM_1 by an order of magnitude. This was expected since it uses much shorter sequences as inputs, and the pairwise-matching algorithm performs faster with shorter sequences. Split times (see logs) show that reconstruction times were negligible and that the few instances of resource limitations coincided with the matching of the longest sequence VioB. Running times were similar for both sequences - between 10 and 12 s.

**TABLE 2:** Performance of the CM_2 Algorithm. Performance was assessed for constructs of opposite complexity and different reconstruction thresholds. Results in the top part of the table are for the minimal QC scenario, and show computation times are unaffected by the constructs and threshold. Results in the bottom part of the table are for the maximal QC scenario, and also show computation times are unaffected by the constructs and threshold.

Minimal quality control with CM_2		
	Threshold = 0.99	Threshold = 0.75
Vio-0000	10.32 ± 0.15 s	10.22 ± 0.14 s
Vio-0007	9.70 ± 0.08 s	9.52 ± 0.33 s
**Maximal quality control with CM_2**		
	**Threshold = 0.99**	**Threshold = 0.75**
Vio-0000	10.71 ± 0.17 s	11.43 ± 0.88 s
Vio-0007	10.20 ± 0.03 s	10.06 ± 0.07 s

#### Final Comparison of the Algorithms

Finally, a test to assess what effects the demands of the Smith-Waterman algorithm have on the performance of all three algorithms was designed. Specifically, the target (the input construct) was increased in size and complexity. Since the reconstruction step of the algorithms is so efficient (repetitions are effectively tackled), we concatenated the construct Vio-0000 with itself twice and thrice. This way a test set of three constructs of increasing lengths and complexity was generated: Vio-0000 ✖1 (8.5 kb, 5 TUs and 20 components); Vio-0000 ✖2 (17 kb, 10 TUs and 40 components), and finally Vio-0000 ✖3 (25 kb, 15TUs and 60 components).

All three algorithms were then applied to all three synthetic constructs - for the minimal and maximal QC scenarios. Since all constructs were ideal (no mutations introduced), the overlapping parameters were set to 0, and detection threshold set to 0.99 (very high). Input data for CM_2 were generated by slicing the constructs TU by TU as in previous tests. Results for these tests are displayed in Table 3.

**TABLE 3:** Comparative performance of the algorithms for constructs of increasing complexity and lengths. Performance was estimated for all three algorithms and in the minimal and maximal QC cases. The test constructs were generated by concatenating the test construct Vio-0000 a number of times.

	Vio-0000 ✖1 8.5 kb	Vio-0000 ✖2 17 kb	Vio-0000 ✖3 25 kb
CM_0	Minimal QC: 36.60 ± 0.73 s	Failure	Failure
	Maximal QC: >30 h (est)	Failure	Failure
CM_1	Minimal QC: 78.70 ± 6.61 s	Minimal QC: 524.04 ± 3.16 s	Minimal QC: 1776.53 ± 4.74 s
	Maximal QC: 109.86 ± 15.10 s	Maximal QC: 663.01 ± 2.88 s	Maximal QC: 2,197.00s ± 3.51 s
CM_2	Minimal QC: 10.32 ± 0.15 s	Minimal QC: 18.86 ± 0.16 s	Minimal QC: 27.72 ± 0.76 s
	Maximal QC: 10.71 ± 0.17 s	Maximal QC: 21.00 ± 0.16 s	Maximal QC: 30.04 ± 0.26 s

The results make plain a crucial limitation of CM_0 (the tests failed due to lack of RAM): its range of application is limited to shorter sequences. CM_1’s performance also suffered for the largest sequences. With Vio-0000 ✖1, full analysis took less than 2 min per construct (a reasonable time for everyday use), but shot up to 10 min for Vio-0000 ✖2, and 35 min for Vio-0000 ✖3 (which would present challenges at scale). This is indicative of the authors’ experience - CM_1 performs well with sequences of less than 10 kb, and becomes expensive for sequences above 15 kb. It is worth noting that when genetic diversity is generated with short regulatory elements, running maximal QC only adds 20% to the run time (matching short sequences remains a cheap operation even for long targets). In everyday use, the run times can be improved by not matching the longer CDS components (or only fragments of them) and focusing on the shorter, regulatory components.

Finally, CM_2 which uses short sequences as inputs remains fast in all cases, and performs linearly with the construct length. This was expected, since reconstruction and slice recombination have negligible cost, and slices take a similar time to be processed.

### Real Life Example—Lycopene Operon

To complement the synthetic benchmarks, a real-world use case - encountered by the Kitney Lab during recent work on lycopene production (Exley et al., 2019) - will now be discussed. All input sequences and analysis outputs can be found at https://github.com/kitneylab/cmatch.

The results are indicative of the kind of real-life performance and challenges for a combinatorial pathway engineering project. In particular, they are instructive of how algorithm parameters must be tweaked to deal with real, imperfect data.

**FIGURE 7:**
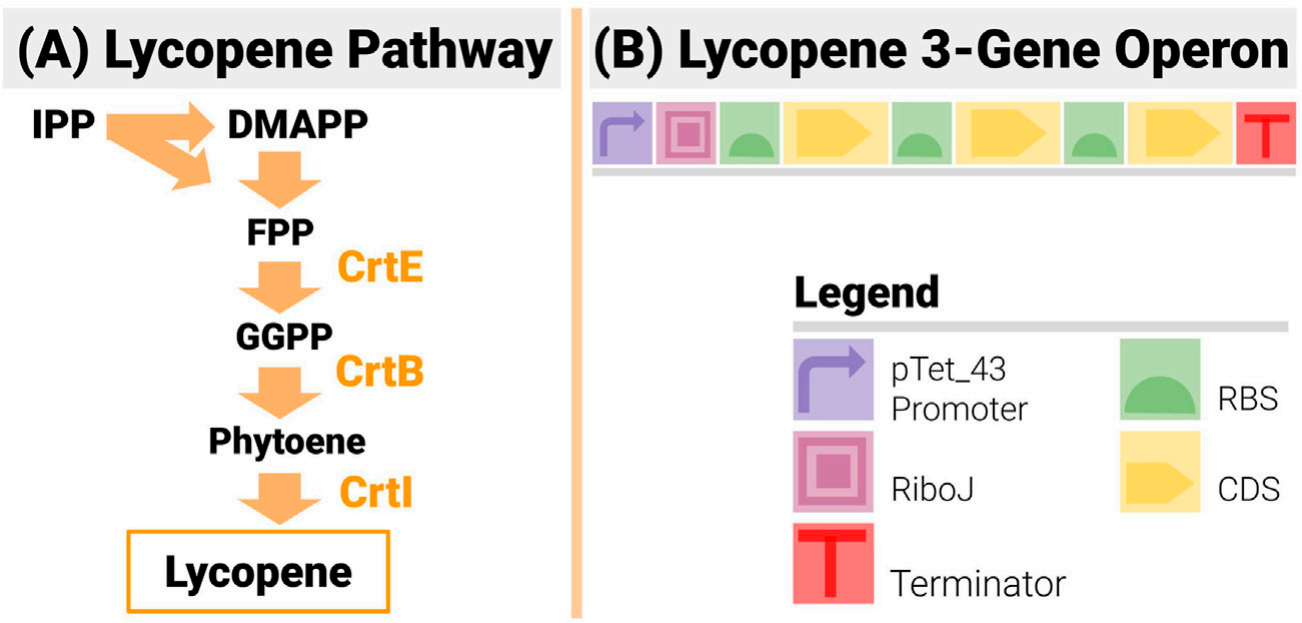
The 3-Gene Lycopene Pathway and its Implementation with an Operon Design. **(A)** The lycopene pathway is made up of only 3 enzymes (CrtE, CrtB and CrtI) - making it a very popular test bed in metabolic and combinatorial pathway engineering. **(B)** The design is made up of 3 genes in the set order CrtE-CrtI-CrtB. An inducible promoter is used to drive the operon and an insulating element RiboJ is inserted post promoter.

Lycopene, a naturally produced bright-red pigment, is a carotenoid present in many plants and organisms (Yamano et al., 1994; Gallego-Jara et al., 2015). Its antioxidant properties make it of high value to the pharmaceutical industry, and a popular colouring agent (Ciriminna et al., 2016). Its chemical synthesis is limited by high cost, low yield and quality (Sevgili and Erkmen, 2019). It is an extremely popular case study in metabolic engineering, as its pathway (Figure 7A) is made of only three enzymatic reactions catalyzed by the enzymes crtE, crtB and crtI (Misawa et al., 1990; Yoon et al., 2006). The construct design (see Figure 7B) was as follows:

● It used an operon design to reduce the design space compared to designs based on transcription units, and limit homologous recombination.
● An inducible pTet_43 promoter drives the operon:
○ The promoter is very tightly shut off in the absence of an inducer to make sure the transformed cells grow unencumbered in the overnight culture.
○ The switch from off to ON to OFF is steep and the “ON” strength of the promoter is high (estimated at RPU = 1.2 from previous iGEM results).
● An insulating RiboJ (Clifton et al., 2018) was inserted post promoter.
● Gene order was fixed to CrtE-CrtI-CrtB (a common order in lycopene studies)
● All three RBS in the operon could be changed. For each position, all 12 RBS from the BioLegio library (RBS-A01 to RBS-A12) were used—yielding a space of constructs with 1,728 members

Quality control was limited by practical limitations that are typical Sanger sequencing. Three reverse primers were therefore designed—all originating a few hundred base pairs into the CDS. CM_2 was used instead of CM_1, since multiple sequences were used as input. Before analyzing the real data, simulations with synthetic data were conducted. As previously, constructs of opposite complexity (in terms of repetition) were used. BASIC assembly was simulated with a dedicated Python module (Haines, 2021). Input sub-sequences were generated by slicing the simulated constructs as if reverse primers originating from the end of the CDS were used - no mutations were introduced. The most extensive form of quality control offered by cMatch was conducted - that is the constructs were matched against their entire design space. In all cases, the constructs were identified with a perfect score of 1. Results from the simulations showed that processing each test construct took around 40 s. All elements to match took between 0.5 and 1.5 s to match, even the CDS (CrtE, I and B being short genes).

Real data for 10 constructs were then analyzed with cMatch. Maximal quality control was conducted for each of them (i.e., they were checked against the entire design space, rather than against themselves). As the primers originated only a few 100 base pairs into the CDS rather than at their ends, input sequences were only a third as short (700 vs. 2,000 base-pairs) as in the test with synthetic data, and processing times for the matching step were faster by an order of magnitude (see logs).

Search parameters such as detection threshold and overlap parameter can be adjusted by the user. They help filter what components may proceed to the reconstruction phase of the algorithm. With reliable sequencing data, it is reasonable to set them up to stringent values (high threshold, no overlap). This was not the case with the lycopene data. To account for poor sequence quality (the start and end of the sequences showed low phred score), the global matching threshold was lowered to 0.5. Authors’ experience with other datasets has been that input sequences are often unreliable at their starts and ends and that one of the most efficient ways to deal with such issues is to lower matching thresholds so poorly-matched components can qualify for the reconstruction steps. Well-matched components still qualify, and step-by-step reconstruction still returns the best candidates - only this time the best candidates include poorly-matched components. Another important and complementary way has been to relax the constraint on component-overlapping (via the epsilon parameter). When the sequence data is corrupted, the pairwise matching algorithm is prone to using insertions and deletions—leading to components that may overlap by a few base pairs in some cases. Increasing epsilon to 3 allowed reconstruction to proceed successfully when identification of the CDS was poor due to sequencing issues.

## Discussion

We have presented a novel, automatic tool, cMatch, to reconstruct and identify a synthetic genetic construct from its sequence, or more practically, a set of sub-sequences. The method is based on two pieces of information: knowledge of its modular structure, and its libraries of components. Although first intended (and developed) for combinatorial pathway engineering problems and lifting their QC bottleneck, cMatch is not restricted to these applications. The algorithms can indeed be applied to any synthetic, modular construct, provided a template and a list of elements for every position of the template are available.

### Applications

cMatch has been designed to perform an operation that we have called “construct-matching,” and which is more thorough than simple sequence-matching or alignment. Construct-matching is concerned with matching sequence data to constructs at the functional level, practically searching for its components, checking if their order matches a given template, and then quantifying for each component how they differ from expected components. Working at component level is important in order to deal with small regulatory components, and eliminate what biases matching methods have towards large sequences. For each component a matching (homology) score is derived. These scores and their weighted average are then used to quantify the reliability of the predictions.

Construct-matching also goes further than automated annotation and sequence alignment, as these matching scores are used to make decisions regarding the matching (reconstruction does not proceed unless all components are identified with sufficient precision, and fails if some components are missing or no component combination can be found that matches the template), and to rank the possible combinations.

To our knowledge no software has been designed to exploit the advantages of modularity in construct design to perform such a task—despite the centrality of the concept in synthetic biology, and the level of friction quality control and similar forms of verification introduce into project workflows when performed at large scale, and the obvious reliability and reproducibility issues associated with these tasks.

As previously stated, cMatch was first intended and developed for combinatorial pathway engineering problems. These applications are subject to a particular set of constraints that require the development of precise, reliable, automated quality control for their constructs—namely the need to deal with the large amount of data generated by these applications and the need for precision in the identification of the small regulatory elements. cMatch is not restricted to these applications however: it can be applied to QC for any synthetic, modular construct, provided a template and a list of elements for every position of the template are available.

Thanks to the way it exploits construct modularity, cMatch is also capable of performing two forms of quality control of opposite complexity (called minimal and maximal QC in this work):

- Minimal QC: This is the lowest and most common form of quality control. It aims to check that the sequencing data correspond to a given construct, or to what degree they differ.
- Maximal QC: Sequencing data are compared to all the constructs in the entire design space at once. This is a more thorough form of QC, as it offers some insurance against practical errors such as mislabelling. It also offers estimates of how more likely the best match is compared to the best other possibilities. This is a useful feature when data can not be assumed to be completely reliable, or to deal with the shortest components, for which changes of a few base-pairs (due to mutations or sequencing errors) may lead to the identification of different library elements.

cMatch can also be applied to other applications than quality control, and to the more general problem of identification of synthetic constructs lying in a specified design space. A typical example of such need is encountered when pooled construction workflows are used. These workflows yield little prior information on the genetic material—except that it matches a given template, was assembled from a known set of components and therefore lies in a, possibly large, space of possibilities. This is insufficient in general, and the link between colonies and their genetic material must be re-established, so phenotypic data may be correlated with genotypic data. This scenario was encountered by the Kitney Lab during its recent work on the lycopene pathway. Because lycopene is toxic due to its propensity to accumulate in cell walls (Taylor and Heap, 2020), the expression of some constructs was hampered - the issue being particularly severe for operon designs driven by constitutive promoters as in (Exley et al., 2019). To identify the viable region of the design space, a novel method to bootstrap a DoE workflow was developed, based on a pooled approach. Cells were transformed with random constructs in a one-pot reaction containing all the different combinations of the parts. Once transformed and grown, viable colonies were selected at random, colony-PCR was performed. Construct identification was a crucial step of the workflow that was not tractable without cMatch.

### Reproducibility, Reliability and Robustness

Reproducibility, reliability and robustness have been at the core of the design of cMatch and its algorithms. Automating quality control (regardless of the way it is accomplished, and what sub-tasks it performs) obviously prevents analysis from being affected by considerations affecting human operatives such as fatigue, waning focus and general human inconsistency. Reproducibility was improved by making the algorithms deterministic with our choice of the Smith-Waterman algorithm as the foundation for cMatch to build on. Only computation times vary for a given input, and search parameters (template, libraries), and do so according to available computing resources—benchmark results in *Results—Applications and Benchmarks* section with the violacein example show limited variation in that regard. Synthetic results from the violacein example show that the algorithms correctly identify the ground truth and return a score of 1.0 for all its components. The algorithms are therefore reliable.

Robustness, defined as “the quality of being strong, and healthy or unlikely to break or fail” (Def. 1. Cambridge Dictionary) is, however, more than just reliability and reproducibility, and extends to other parts of the construct-matching process - the most important being decision-making in the face of uncertainty. Unlike the tests with synthetic data, it is impossible in practice to be sure that an input sequence is itself reliable and to be fully trusted. Errors can be either due to sequencing or be indicative of a genuine alteration of the genetic content caused by a few mutations, some more likely to alter construct output than others, or at the other end of the spectrum proof of assembly failure).

cMatch’s algorithms offer several mechanisms to deal with uncertainty in input data.

The first mechanism helps deal with poor data: users can lower the constraints for a successful analysis—practically, the constraints on component-matching (matching threshold) and reconstruction (admissible overlap). Benchmarking results show the cost of relaxing these constraints to be minimal thanks to the efficient step-by-step reconstruction.

The second mechanism is through quantification. Any non-perfect matching score can be used as a warning flag warranting further investigation of the construct or some of its components (a level of analysis unobtainable without a component-based software). Further investigation can take several forms. It can be a simple repetition of the sequencing process (in case the best match is close to the input sequence and it is legitimate to suspect a sequencing error because discrepancies are located in regions with low phred score) or a more drastic decision to repeat the construction. In either case, construct-matching can be repeated on new sequence data and the best prediction, as measured by the overall matching score (or any user-defined function on the component scores), can be retained as the most reliable output. Previous estimates of construct properties may then need adjusting - for instance a new estimation of the translation rate can be computed if mutations are found in a UTR region and it is concluded the construct has mutated.

Quantification allows the estimation of the distance between the best match and all the other constructs in the same design space - thus offering a method to rate how likely the best match is compared to these other possibilities. This is the idea behind what we called maximal QC. Depending on the choice of elements in libraries, some search spaces will yield more conclusive identifications than others, since their constructs are spread further from each other. This should come as no surprise, since manual sequence alignment is notoriously harder and error-prone when libraries contain elements close to each other, for instance differing by a few base pairs after they were generated by random-PCR.

### Practical Considerations

cMatch’s algorithms have been developed to be fast enough for common applications, but also so they do not require special hardware to run on. The benchmarks in *Results—Applications and Benchmarks* section show, analyzing a sequence for a typical problem (constructs with a few genes, search spaces made of a few thousand constructs) only takes a few minutes. Processing a batch of sequences (a few hundreds for QC), will in no way represent an obstacle for a project.

Algorithm inputs have also been chosen to be as intuitive as possible, and so little data conversion must be undertaken before analysis is run. Input sequences are in the .seq format typical of Sanger sequencing, or simple txt format. The search parameters are accessible to the user, and encoded in the human-readable format JSON. The corresponding file has a simple structure that can be easily modified (examples of valid JSON files can be found at https://github.com/kitneylab/cmatch) so data analysis may start soon after the sequences for the components are gathered. Finally, the common txt, Genbank, and SBOL formats are supported for the sequences of the elements in the component libraries.

The most important consideration for the choice of the construct-matching algorithms relates to the nature of the input - whether a single or multiple sequences are used. This choice is in general a direct consequence of the sequencing method. CM_2 is the default algorithm for multiple inputs. It is worth noting that because sequences used for inputs will be short, some limitations of the Smith-Waterman algorithm will have limited impact on performance. CM_1 remains the algorithm to use when a single sequence is used as input, as would be the case with NGS sequencing.

An important feature of both CM_1 and CM_2 is that they were built on top of the Smith-Waterman algorithm for pairwise matching. This algorithm is extremely reliable, but its performance suffers with long sequences. When possible, shorter component sequences should be used - for instance with CDS or hybrid components. Benchmark tests have shown that the performance of CM_1 degraded with input sequences longer than 10 kb - so would CM_2 if such a long sequence featured in its inputs. Splitting strategies are also being investigated to reduce the size of the inputs and speed up computations at the component-matching step of the algorithms.

Both CM_1 and CM_2 rely on the availability of two specific pieces of information.

They first rely on the availability of the construct template. Algorithms for the purpose of sequence investigation (and template identification) are outside the scope of this work—construct investigation being of little immediate use to the Kitney Lab in its everyday workflows. They offer useful capacities however, for instance as part of an audit of resources in freezer storage. Variants of CM_1 and CM_2, that modify the reconstruction phase so all combinations are pruned not against a set template but under a set of positional and grammatical rules, will be investigated in subsequent works.

They also need a full list of the elements in the component libraries, since they are based on pairwise-matching of known sequences. In case of missing elements, a possible strategy is to use a very large library of components from an external database, lower the pruning thresholds for these components, and match the large libraries against the target sequence. The best matches can then be used to locate the missing elements providing they have enough in common. Because of the high degree of parallelism in the algorithm, we are confident that analysis of a sequence will remain tractable. For longer components, the faster but less reliable BLAST search may be recommended.

## Conclusion

cMatch is a simple, lightweight tool to perform quality control of modular synthetic constructs at speed and scale. Although originally developed for the application case of combinatorial pathway engineering and making QC frictionless in optimization workflows, cMatch can be used in many different settings - we leave it to the reader to adapt this versatile tool to their own applications. The use of cMatch has made a significant difference to the operation of the Kitney Lab, and given a significant boost to their productivity and to their confidence in the reproducibility of their results. We are confident other adopters will enjoy similar benefits.

### Tool Presentation

cMatch has been implemented in Python 3.9 and is publicly available as an open-source package on the Kitney Lab Github page (https://github.com/kitneylab/cmatch) under MIT license (https://choosealicense.com/licenses/mit/). The core functionalities are implemented as three different modules: matching.py, reconstruction.py and extension.py which respectively implement the core Sequence, Component Libraries and Component classes and their matching methods (calling biopython pairwise2 local alignment function), the reconstruction and extension functions. All input and output files are in JSON (for simplicity) except the sequence files (in .seq). The CMatch package implements the CM_1 algorithm to analyse a single sequence, and its CM_2 extension for multiple sequences.

## Data Availability

The datasets presented in this study can be found in online repositories. The names of the repository/repositories and accession number(s) can be found in the article/Supplementary Material.
