# Supplementary material for: Removing the Bottleneck: Introducing cMatch - A Lightweight Tool for Construct-Matching in Synthetic Biology
Source: Front Bioeng Biotechnol. 2022 Jan 10;9:785131. doi: 10.3389/fbioe.2021.785131 (PMC8784771; doi:10.3389/fbioe.2021.785131)
Supplement: Supplementary file 1 [file DataSheet1.PDF]

Supporting Information

Removing the Bottleneck: Introducing cMatch - a Lightweight Tool for Construct-Matching in Synthetic Biology

Authors:

Alexis Casas, Matthieu Bultelle, Charles Motraghi, and Richard Kitney ∗

Authors' Institutional Affiliation

Department of Bioengineering, Imperial College , London

Exhibition Road , London SW7 2BX UK

Corresponding E-mail: r.kitney@imperial.ac.uk

1 - Tool Presentation

The matching software cMatch has been implemented in Python 3.9 and is available as a package on the Kitney Lab Github page [https://github.com/kitneylab/cmatch]. The core functionalities are implemented as three different modules: `matching.py`, `reconstruction.py` and `extension.py` which respectively implement the core Sequence, Component Libraries and Component classes and their matching methods (calling biopython pairwise2 local alignment function), the reconstruction and extension functions.

The tool takes different input files:

- Template file of the construct (JSON)
- Target file containing the target names and slices (JSON)
- The DNA sequences to analyse (.seq)

and outputs different matching result files as well as a log file (optional) containing a detailed timing of the different functions:

- Matching results file (JSON) containing the candidate parts and their scores and starting/ending alignment positions
- Reconstruction results file (JSON) containing the reconstructed paths of the different slices
- Extension results (JSON) the final reconstruction of the construct from the different slices.

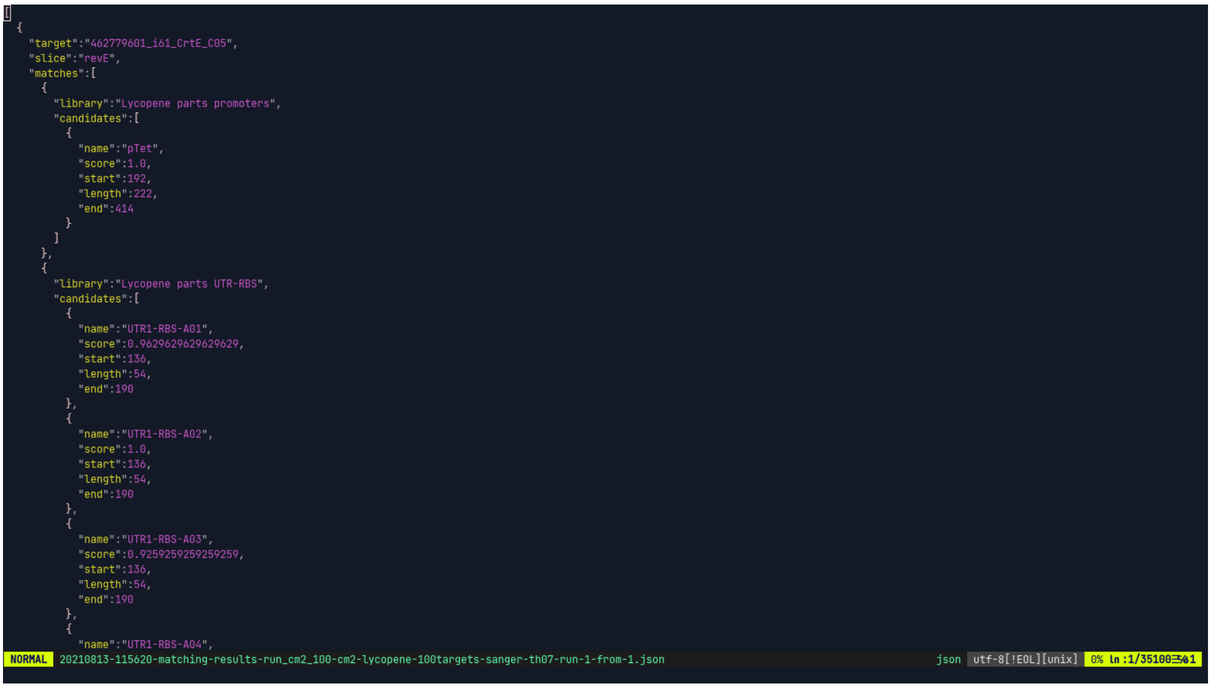


```
[
  {
    "target":"462779601_i61_CrtE_C05",
    "slice":"revE",
    "matches":[
      {
        "library":"Lycopene parts promoters",
        "candidates":[
          {
            "name":"pTet",
            "score":1.0,
            "start":192,
            "length":222,
            "end":414
          }
        ]
      },
      {
        "library":"Lycopene parts UTR-RBS",
        "candidates":[
          {
            "name":"UTR1-RBS-A01",
            "score":0.9629629629629629,
            "start":136,
            "length":54,
            "end":190
          },
          {
            "name":"UTR1-RBS-A02",
            "score":1.0,
            "start":136,
            "length":54,
            "end":190
          },
          {
            "name":"UTR1-RBS-A03",
            "score":0.9259259259259259,
            "start":136,
            "length":54,
            "end":190
          },
          {
            "name":"UTR1-RBS-A04",
NORMAL 20210813-115620-matching-results-run_cm2_100-cm2-lycopene-100targets-sanger-th07-run-1-from-1.json    json  utf-8[!EOL][unix]  0% ln :1/35100≡:1
```


Figure S1: Example of a matching results JSON file for the Real Life Example of the Lycopene Operon (see full JSON in the supplementary files)

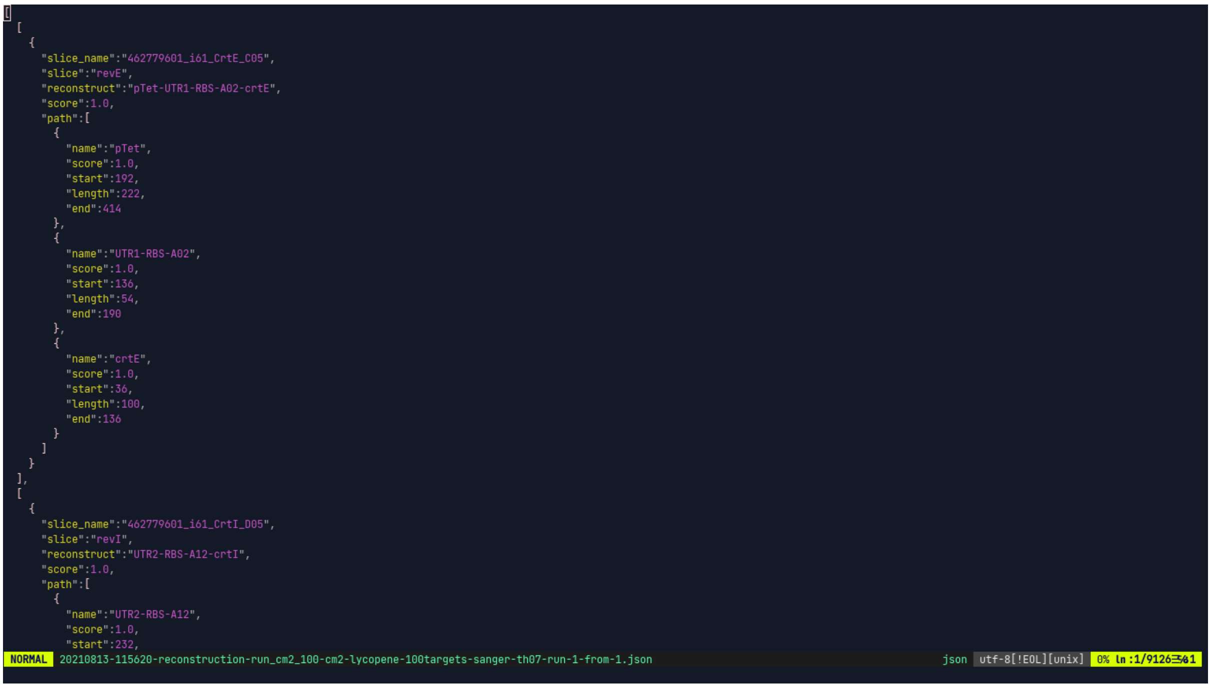


```
[
  [
    {
      "slice_name":"462779601_i61_CrtE_C05",
      "slice":"revE",
      "reconstruct":"pTet-UTR1-RBS-A02-crtE",
      "score":1.0,
      "path":[
        {
          "name":"pTet",
          "score":1.0,
          "start":192,
          "length":222,
          "end":414
        },
        {
          "name":"UTR1-RBS-A02",
          "score":1.0,
          "start":136,
          "length":54,
          "end":190
        },
        {
          "name":"crtE",
          "score":1.0,
          "start":36,
          "length":100,
          "end":136
        }
      ]
    }
  ],
  [
    {
      "slice_name":"462779601_i61_CrtI_D05",
      "slice":"revI",
      "reconstruct":"UTR2-RBS-A12-crtI",
      "score":1.0,
      "path":[
        {
          "name":"UTR2-RBS-A12",
          "score":1.0,
          "start":232,
NORMAL 20210813-115620-reconstruction-run_cm2_100-cm2-lycopene-100targets-sanger-th07-run-1-from-1.json    json  utf-8[!EOL][unix]  0% ln :1/9126≡:1
```


Figure S2: Example of a reconstruction results JSON file for the Real Life Example of the Lycopene Operon (see full JSON in the supplementary files)

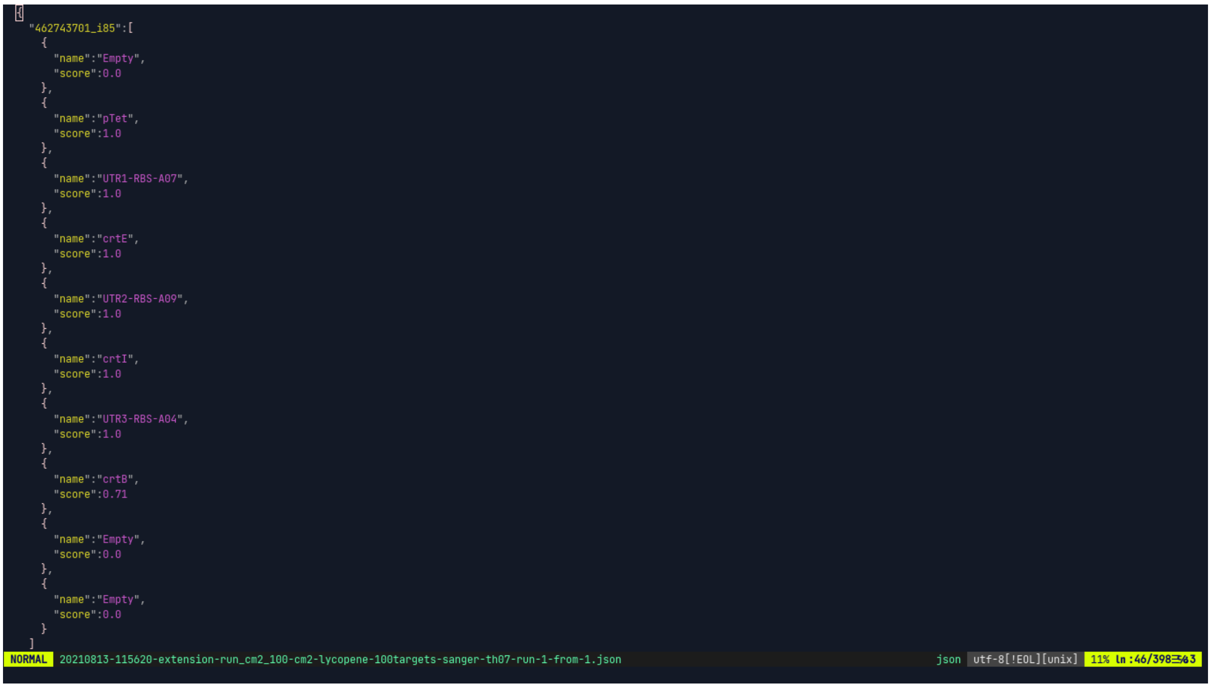

```json
{
  "462743701_i85":[
    {
      "name":"Empty",
      "score":0.0
    },
    {
      "name":"pTet",
      "score":1.0
    },
    {
      "name":"UTR1-RBS-A07",
      "score":1.0
    },
    {
      "name":"crtE",
      "score":1.0
    },
    {
      "name":"UTR2-RBS-A09",
      "score":1.0
    },
    {
      "name":"crtI",
      "score":1.0
    },
    {
      "name":"UTR3-RBS-A04",
      "score":1.0
    },
    {
      "name":"crtB",
      "score":0.71
    },
    {
      "name":"Empty",
      "score":0.0
    },
    {
      "name":"Empty",
      "score":0.0
    }
  ]
NORMAL 20210813-115620-extension-run_cm2_100-cm2-lycopene-100targets-sanger-th07-run-1-from-1.json    json utf-8[!EOL][unix] 11% ln :46/398≡%3
```

Figure S3: Example of an extension results JSON file for the Real Life Example of the Lycopene Operon (see full JSON in the supplementary files)

2 - CM_1 - Construct Reconstruction

CM_1 relies on an step-by-step method to control combinatorial explosion and prune out paths as soon as they break template constraints The need for such a reconstruction method can be illustrated with the violacein construct Vio-0000 - a construct with 5 identical RBS (all B0030) - designed as shown in Figure S-4.

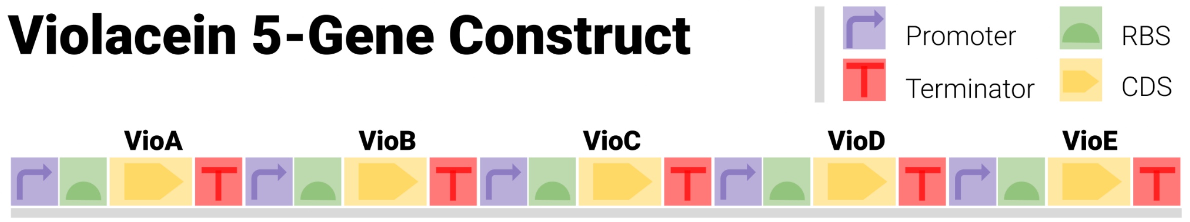

Figure S4 - The 5-Gene Violacein Pathway and its Implementation with a TU-Design.
(A) The design is made up of 5 transcription units (hence 20 components) in a set gene order. All units share the same promoter (BBa_J23101) and terminator (BBa_B0015) - providing a stiff test for the algorithm, due to the large number of potential repetitions.

After the first step of the algorithm, and with a high threshold of 0.99:

- Component 1: Promoter J23101 is located in 5 possible locations (one being early in the sequence, which is the correct location, and four in further incorrect locations.
- Component 2: RBS B0030 is located in 5 possible locations (one being early in the sequence, which is the correct location, and four in further incorrect locations.
- Component 3: CDS VioA is located in 1 possible (and correct) location only.
- Component 4: Terminator B0015 is located in 5 possible locations (one being early in the sequence, which is the correct location, and four in further incorrect locations.
- See Logs for more information on the further components

With a lower threshold of 0.75, 2 RBS are identified instead of 1, in each of the five RBS positions. Without any attempt to control the combinatorial explosion, the first choice of threshold leads to number of combinations of the order of 10^{11} and 10^{12} for the lower threshold. Pruning against the template (FigureS5-A) is wholly unrealistic.

CM_1's reconstruction method is based on a simple remark: if a construct breaks the template constraints because its n-th component overlaps one of the previous components (positions 1 to n-1), then all constructs with the same first n components break the template constraints and need pruning. It follows reconstruction can be efficiently implemented through the iterative construction (Figure 5-B) of a layered directed acyclic graph, where each layer of the graph corresponds to a position in the template, the nodes for a given layer are the components identified in Step1 for that template position, and directed edges link components of consecutive layers if and only if they fulfill the positional requirements.
Rather than using a graph structure, CM_1 uses lists. The cost in terms of memory (a list of paths takes more space than a graph) is offset by the simplicity of the list implementation (especially by the gain of readability and ease of pruning of branches).

Practically, reconstruction takes place in template order, and builds all possible paths one step at a time as follows:

- Iteration 0 : paths[0] = [] Empty List
- Iteration N:
  - Path Expansion : new_paths = paths[N-1] x Can[N] , where paths[N-1] is the list of paths after iteration N-1, Can[n] is the list of candidate components, returned by step 1 of the algorithm, at template position N, and x is the combinatorial product (Cartesian list product)
  - Path Pruning: pruned_paths = prune(new_paths). For a path to pass the pruning phase, the newly-added component must be located downstream of all the previous components - it is enough that the new component is located downstream of the component in position N-1 since the paths are generated iteratively. In our implementation, an overlapping parameter epsilon is introduced to specify how many base-pairs adjacent components can share.
  - Returning Results : paths[n] = pruned_paths.

Step-by-step reconstruction proceeds as follows for Vio-0000:

- Step 1: There are 5 possible paths, made of the same promoter, in different locations
- Step 2: To each of the previous 5 possible paths, the 5 possible UTRs are added - leading to 25 possible paths. Positional constraints (the UTRs must be located further than the promoters) eliminate most paths - leaving only 15 paths (5 paths with the promoter in its earliest location, 4 with the promoter in its second position etc..).
- Step 3: to each of the previous 15 paths, the only possible CDS is added. Since the location of the CDS is known and is unique, this only leaves one path possible.
- Step 4: to the only path left, the 5 possible terminators are added - all paths are compatible and passed on to the next step.
- And so on until the whole construct is reconstructed (and only one candidate is left)

With lower detection thresholds, the number of paths increases several-fold but remains of the order of the number of paths reconstruction would have to deal with, if the components were only identified in single locations (this number is the baseline for the reconstruction). For instance if the threshold on the UTRs was lowered so 2 UTRs had a matching score above the threshold, then the first step of the algorithm would return 10 possibilities for component 2 (2 UTRs, in 5 possible locations). After the first step of the reconstruction there would be 5 paths; 30 paths after the second, 2 after the third, 10 after the fourth etc…. Eventually there would be 32 possible constructs left - to be ranked according to the overall matching score derived from the scores of their individual components.

Results in figure S6 illustrate how the step-by-step reconstruction controls the combinatorial explosion related to component repetition. Results are displayed for two cases, with the target construct Vio-0000. In the first scenario (Figure 6 - A) , a high threshold (0.99) is used and only one element can be identified per library (but in the case of promoters, UTRs and terminators, in 5 distinct locations). In the second scenario (Figure 6 - B), a lower threshold of 0.75 was chosen so this time 2 possible UTRs are detected for each of the 5 locations. For each scenario, the number of paths vs the step of the step-by-step reconstruction is plotted for three different cases:

- Without pruning to control the combinatorial explosion - yielding a number of paths of the order of 10^{11} in the first scenario and 10^{12} in the second.
- With pruning - yielding a number of paths of 20 maximum in the first scenario and 80 maximum in the second.

- The baseline number of paths - the number of paths if there was no uncertainty on the component locations (and the limit case for the reconstruction)

With the step-by-step reconstruction and the associated pruning, the number of paths stays close to the baseline scenario - and ends up with the same number of candidates, whereas the no-pruning scenario sees the number of paths explode.

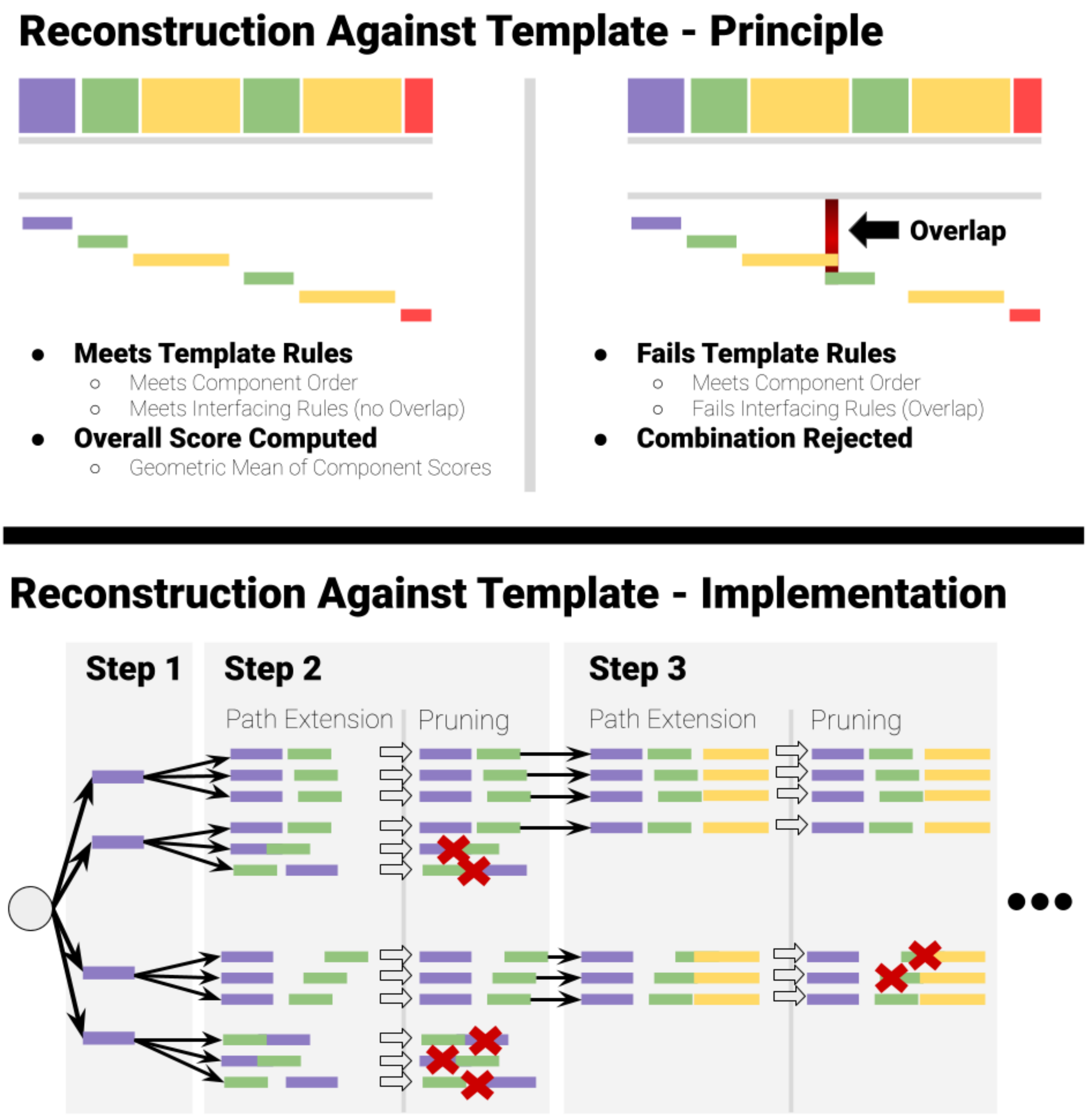

Figure S5 - Reconstruction against the template - principle (A) and implementation (B)

(A): On the left hand side, components are assembled in a way that matches the template (same order, admissible overlapping). On the right-hand side, the reconstruction is not rejected since two components overlap by more than the allowed margin epsilon. (B): Only the first three steps of the reconstruction iteration are shown. First, 4 possible promoters are added to an empty path. Then 3 UTRs are added; 5 out of 12 paths are pruned. A CDS is added and 2 paths are pruned - leaving only 5 possible paths after the first 3 steps.

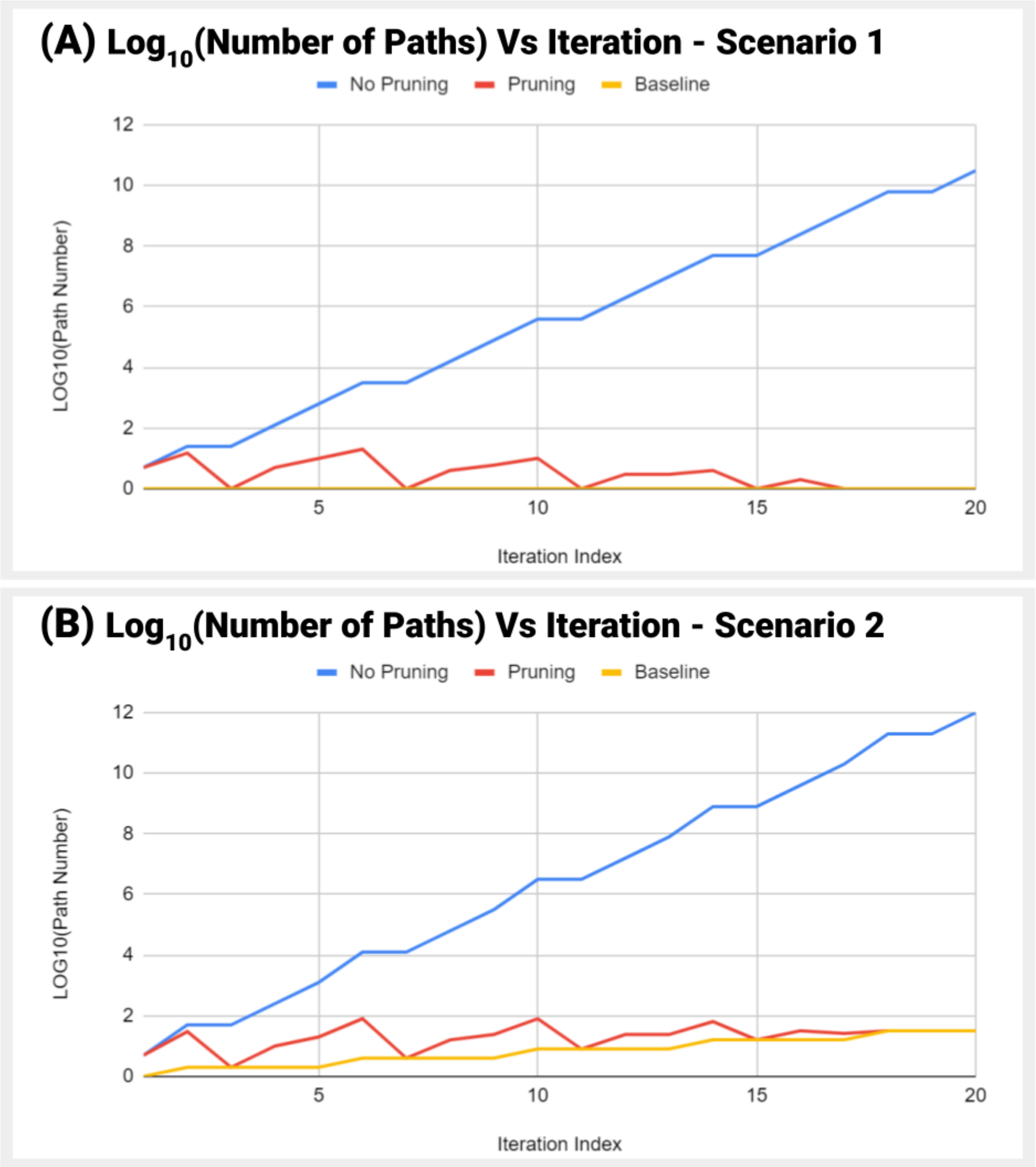

Figure S6 - Controlling the Combinatorial Explosion thanks to the step-by-step reconstruction.

All results are obtained with the target construct Vio-0000 (which is the most challenging construct for reconstruction due to its use of 5 identical promoters, 5 identical UTRs and 5 identical terminators. (A): a high threshold (0.99) is used and only one element can be identified per library (but in the case of promoters, UTRs and terminators, in 5 distinct locations). (B): a lower threshold of 0.75 is used and this time 2 possible UTRs are detected instead of 1 (as in the first scenario, 5 locations are possible).

3 - CM_1 - Pseudo-Code Implementation

Let T be the template. And S be the input Sequence.
We let L be the set of all libraries $L = (L_i)_{i=1....N}$.. Our implementation uses a list in the JSON template file, and orders the libraries as per the template. The index i corresponds to the ith position in the template and , by convention, starts at 1.
The library L_i (also practically implemented by way of a list in the JSON template file) contains all the possible elements for the ith component of the template $L_i = (e_{i,j})_{j=1...K(i)}$
Where K(i) is the size of the library Li (i.e the number of elements it contains). It is assumed that all elements in the library are distinct.

Initialisation

```
NB_MATCHES = 5                                  # best 5 constructs are returned
LIBRARY_THRESHOLDS = [0.9,0.9,.....,0.9]        # Library thresholds
EPSILON = 3                                     # Tolerance parameter
```

3 Sets of parameters are defined prior to application of the algorithm:

- NB_MATCHES: the number of constructs the algorithm returns - by default it is set to 1 (the algorithm then only returns the construct with the highest score)
- LIBRARY_THRESHOLDS: each library is assigned a minimum matching threshold. This threshold is used at the end of Step 1, to prune the components and only retain the components with the best scores.In our implementation, all thresholds are stored in a list ordered according to the template, and are set to 0.9 by default
- EPSILON: components interfacing is defined by the maximum number of base pairs successive components may share. By default a value of 0 is used (no overlap)

In this pseudo-code we use the following functions (their implementation is not shown)

- Match_Element is the implementation of the pairwise alignment algorithm (Smith-Waterson algorithm, Biopython implementation). For a given input tuple (element, Sequence) , the function searches for the element in the input sequence and returns a tuple (score,start,end), where:
  - score is the matching score for the element
  - Start and end are the start and end indices of the element in the input sequence.
- Check_Path_Validity checks whether a path is admissible and returns a boolean. It has two inputs: the path to check and the tolerance EPSILON. In our implementation, the function only checks the relative positions of the last two elements of the path is admissible, that is for a path of length p, encoded as a list with indices 0 to p-1:
  - Before_last = path[p-2] = (element_bl,start_bl,end_bl)
  - Last = path[p-1] = (element_l,start_l,end_l)
  - The boolean admissibility condition then is (start_l-end_bl+EPSILON≥0)
  - By default, a path of length less than 2 is admissible
- Compute_Path_Score calculates the overall matching score over a reconstruction path - in our python implementation, the geometric mean of the matching scores of the components in the path is used (using the geomean function in the numpy module).
- Sort_Constructs_by_Score is a function that sorts a list in descending order according to the construct score - in our python implementation it is based on the sorted function and uses lambda x:x[1] as key, as it is applied to (construct, score) tuples.

Step 1 - Component Matching

```
Matched_Lists = []
For i,library in enumerate(L):                # Loop over all libraries in L
        threshold = LIBRARY_THRESHOLDS[i]     # Matching Threshold

        Matched_Elements = []
        For element in library:
                (score,start,end) = Match_Element(element, S)
                If score > threshold :
                        Matched_Elements.append((element,score,position))
                        # element  selected as its score is high enough
        Matched_Lists.append(Matched_Elements)
Return Matched_Lists

# Matched_Lists contains the components to be assembled in the next step
# It is a list of lists - the sublists are ordered in the same way as the libraries in L
# hence in template order
```

Step 2 - Reconstruction

```
# Second step : reconstructs all the admissible constructs from the components
# identified in Step 1

# Initialisation
Paths = []                      # Paths contains the results of the successive iterations
Paths[0] = []                   # Initialisation of the Reconstruction
N = len(Matched_Lists )         # the number of components in the template

# Start Iteration
For i,library in enumerate(Matched_Lists ):       # Loop over Matched_Lists
        New_paths = [ path+[e] for path in Paths[i] for e in library]
        # Creating new paths by adding the components at position i+1

        Paths[i+1] = [path if Check_Path_Validity(path,EPSILON)]
        # Path Pruning
# End Iteration
# Paths[N] contains all the admissible paths of length N
# that is, all the admissible reconstructions

# All that remains is to compute the score of the admissible constructs
All_Admissible_Constructs =
                        [(path,Compute_Path_Score(path) for path in Paths[n]]
# Computes the overall matching score of the admissible constructs
# and appends it to the path
Return All_Admissible_Constructs
```

Step 3 - Returning the Best Matches

Final step : ranks the admissible constructs and returns the best matches

Sorted_Constructs = Sort_Constructs_by_Score(All_Admissible_Constructs)
Sorts the constructs according to their score (high to low)
***Best_Matches* = Sorted_Constructs[0:NB_MATCHES]**
Return Best_Matches

Only the NB_MATCHES with the highest scores are returned

4 - CM_2 - Operations on the Template

For CM_2 to be applicable, each subsequence must have its own modular (sub-)template and these subtemplates must be easily recombined against the main template. The partition and combine steps are efficiently implemented on the template structure presented in Section 2.1 of the paper with a few simple operations. Both operations are demonstrated on a 2-gene operon in Figure S7:

- Data partition uses a simple slicing operation (Figure S7 - A). No concomitant slicing needs to be conducted on the list of libraries in the sourcing portion of the JSON file, thanks to the link contained in the Component sub-schema and pointing to the correct sources.
- Recombination (Figure S7 - B) uses a template addition:
    - Fragment reconstructions are cast back to the complete template - by default missing components are assigned a null score and an empty sequence.
    - Addition takes place component by component; for each component the element with the highest score is chosen. Addition can thus exploit potential redundancy in sub-templates to enhance the reliability of the overall process.

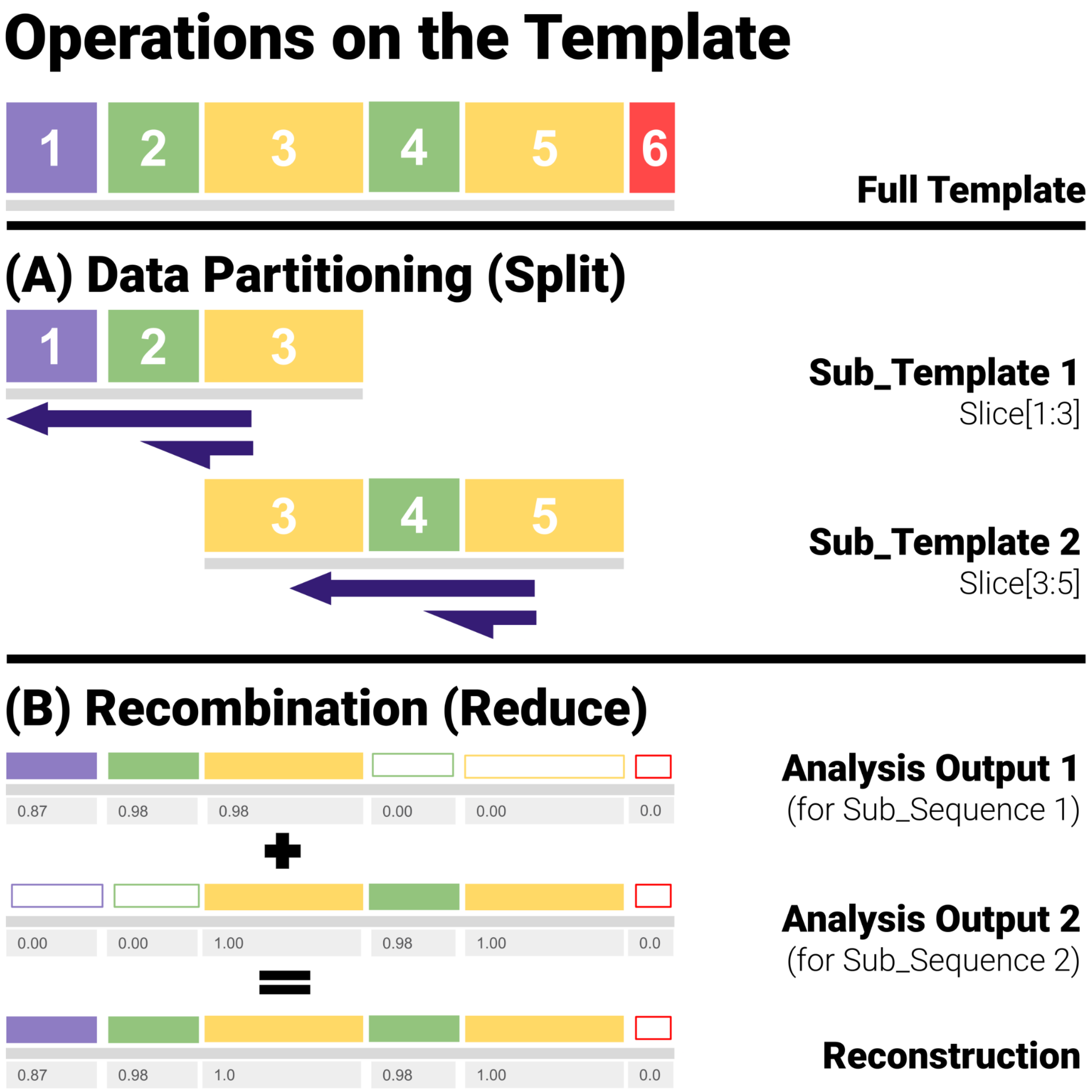

Figure S7 - Operations on the Template : Split and Recombination
(A) Splitting: Two reverse primers are used to generate two overlapping sub-sequences; the sub_templates are obtained by slicing the list containing the components of the template. (B) Recombination: The two sub-templates overlap in the first CDS region, yielding two estimates for that CDS. In the first step of the recombination the results are cast back into the complete template. Missing components are indicated by their hollow structure (and a score of 0). Then the two objects are added - for 4 out of 6 components, addition is straightforward. In position 3 (the first CDS), 2 elements are possible and the element with the better score is retained, as it is deemed the best available estimation. For the last component (position 6, Terminator) the recombination returns an empty component - a direct consequence of the design of the primers.

5 - Running the tests for cMatch

Prerequisites

You need **Python 3.9** and **pip** the package installer for Python https://pip.pypa.io/en/stable/

1. Install a virtual environment (optional):

```
$ python3 -m venv --prompt cmatch venv
```

This creates a virtual environment

2. Activate the virtual environment (optional, only if step 1 was done)

```
$ source ./venv/bin/activate
```

Install the dependencies

1. Clone the repository:

```
$ git clone https://github.com/kitneylab/cmatch.git
```

2. Install the dependencies with pip:

```
$ pip install -r requirements.txt
```

How to run the tests

All the tests are listed in the `supplementary/README.md` file on the Github Repository. There is one python script per test. To launch a test, just execute the corresponding python script as in the example below

How to run the tests for algorithm CM_0:

```
$ python Testing_Algorithm_CM_0.py
```
